# Activation of connexin hemichannels enhances mechanosensitivity and anabolism in disused and aged bone

**DOI:** 10.1172/jci.insight.177557

**Published:** 2024-12-06

**Authors:** Dezhi Zhao, Chao Tu, Lidan Zhang, Teja Guda, Sumin Gu, Jean X. Jiang

**Affiliations:** 1 Department of Biochemistry and Structural Biology, University of Texas Health Science Center at San Antonio (UTHSCSA), San Antonio, Texas, USA.; 2 School of Medicine, Northwest University, Xi’an, China.; 3 The Second Xiangya Hospital, Central South University, Changsha, Hunan, China.; 4 Department of Biomedical Engineering and Chemical Engineering, University of Texas at San Antonio, San Antonio, Texas, USA.

**Keywords:** Bone biology, Bone disease, Drug therapy

## Abstract

Mechanical loading, essential for bone health, promotes bone formation and remodeling. However, the positive response diminishes in cases of disuse and aging, leading to bone loss and an increased fracture risk. This study demonstrates that activating hemichannels (HCs) using a connexin 43 (Cx43) antibody, Cx43(M2), in bone osteocytes revitalizes aging and disused bones. Using a hindlimb suspension (HLS) disuse model and a tibial mechanical loading model, we found that Cx43(M2) inhibited bone loss and osteocyte apoptosis induced by unloading in 16-week-old adult mice. Additionally, it enhanced bone mass in response to tibial loading in 22-month-old aged mice. The HC opening released bone anabolic factor prostaglandin E_2_ (PGE_2_) and suppressed catabolic factor sclerostin (SOST). This suppressed the increase of cortical bone formation and reduction of bone resorption during unloading and promoted trabecular and cortical bone formation during loading. Cx43(M2)-induced HC opening, coupled with PGE_2_ release, effectively rescued unloading-induced bone loss and restored the diminished anabolic response of aged bones to mechanical loading. Activating HCs with the Cx43 antibody holds promise as a de novo therapeutic approach, as it can overcome the limitations of existing treatment regimens for treating bone loss and osteoporosis associated with aging and disuse.

## Abstract 2

Activating mechanosensitive connexin 43 hemichannels with a monoclonal antibody reduces bone loss and reverses impaired bone formation, offering a novel osteoporosis treatment strategy.

## Introduction

Osteoporosis is a prevailing skeletal disease characterized by decreased bone strength and an increased risk of fragility fractures (1). Two marked contributors to osteoporosis and bone loss are disuse and aging. Disuse refers to reduced physical loading on the bones, while aging is associated with a diminished sensitivity of bone cells to mechanical stimulation. Disuse includes prolonged bed rest, physical inactivity, or even space flight, which can lead to a decline in bone mass, osteopenia, and ultimately osteoporosis (2, 3). In the case of aging, bones lose their ability to effectively sense and respond to mechanical stimuli, resulting in an imbalance in bone remodeling characterized by reduced bone formation and increased bone resorption (4). This phenomenon is clinically observed as reduced responsiveness to skeletal loading, which is known to increase bone mass in young adults but often proves ineffective in older adults (5).

Osteocytes comprise the majority of bone tissue cells and are considered primary mechanosensory cells in the adult skeleton (6). Osteocytes have been shown to predominantly express the membrane protein connexin 43 (Cx43) (7). In addition to forming gap junction channels that mediate intercellular communication, Cx43 forms hemichannels (HCs) in osteocytes that allow the passage of small anabolic molecules (≤1.2 kDa) between the intracellular and extracellular microenvironment (8).

Cx43 HCs are highly responsive to mechanical stimulation in osteocytes, leading to the release of small molecules such as prostaglandin E_2_ (PGE_2_) and ATP (9, 10). Our previous in vitro studies have indicated that Cx43 HCs participated in the response to mechanical disuse. Gravity changes during parabolic flight were found to decrease the expression of Cx43 in osteocytes (11). Intriguingly, simulated microgravity using a random position machine (RPM) increased the activity of Cx43 HCs and the release of PGE_2_ (12). The RPM model in vitro stimulates microgravity by “confusing” gravity cells (such as osteocytes, as in the referred study) from perceiving a consistent direction of gravity. This simulation of weightlessness itself is a form of mechanical stimulus, which differs from the hindlimb suspension (HLS) model that directly mimics the “disuse” condition. Our previous in vivo study demonstrated that enhanced Cx43 HCs protect against osteocyte apoptosis in cortical bone during mechanical unloading (13). Osteoblast/osteocyte-specific Cx43–conditional knockout (Cx43-cKO) mice driven by the 2.3 kb *Col1α1* promoter (14) and *Bglap2* promoter (15, 16) have shown that Cx43 deficiency protects against bone loss induced by unloading. However, it should be noted that Cx43 deficiency affects not only Cx43 HCs but also Cx43 gap junctions and Cx43 protein retained in the cytoplasm of the osteocytes (1). As a result, it has remained largely unclear whether the responses observed in the KO models could be solely attributed to Cx43 HCs.

Our previous in vitro studies have also demonstrated that fluid flow shear stress (FFSS) activated the PI3K/AKT signaling pathway, resulting in increased interaction between integrin α5 and Cx43 HCs in osteocytes (17, 18), ultimately leading to the opening of Cx43 HCs (19). The opened Cx43 HCs release the bone anabolic factor, PGE_2_ (10, 20). PGE_2_ acts in an autocrine/paracrine manner, promoting transcriptional regulation of Cx43 (21) and blocking glucocorticoid-induced osteocyte apoptosis (22). Recently, our in vivo studies using transgenic mouse models expressing Cx43 dominant negative mutants in adult mice have demonstrated the crucial role of osteocytic Cx43 HCs and PGE_2_ release in mediating the bone anabolic response to mechanical loading (23–25). Furthermore, the deletion of integrin α5 in osteocytes compromises Cx43 HC function and reduces bone’s anabolic response to mechanical loading (23). Notably, mechanosensitivity and the anabolic response are greatly diminished with aging (26, 27). In aged mice, the expression of Cx43 in osteocytes is markedly decreased (28), and the surviving osteocytes exhibit impaired mechanotransduction (29). Similarly, osteoblast/osteocyte-specific Cx43-cKO mice driven by the 2.3 kb *Col1*α*1* promoter showed an attenuated tibial endosteal osteogenetic response to mechanical loading (30, 31). Given the crucial role of the Cx43 HCs in sensing and responding to mechanical loading, it is speculated that the diminished presence of Cx43 HCs in osteocytes attenuates mechanical responses in aged bone.

Based on previous evidence, we hypothesized that the compromised mechanosensitivity in disused and aged bone resulted from impaired Cx43 HCs. To test this hypothesis, we utilized a monoclonal Cx43(M2) antibody developed in our laboratory (32). This antibody binds to the second extracellular loop domain of the Cx43 molecule. Cx43(M2) is a gain-of-function antibody, distinct from the Cx43(M1) HC-blocking antibody also previously developed by our laboratory (24). Cx43(M2) acts as a Cx43 HC activator that triggers ATP release without altering gap junction channel activity (32). Our results demonstrate that treatment with Cx43(M2) enhanced Cx43 HC activity, resulting in the release of PGE_2_. This treatment protected against unloading-induced bone loss and osteocyte apoptosis in adult mice and improved the anabolic response of the tibial endosteal bone to mechanical loading in aged mice. Considering the lack of effective anabolic therapies for treating aged and disuse-related osteoporosis, the activation of Cx43 HCs, in conjunction with moderate mechanical stimulation, holds promise as a potential therapeutic approach for patients with compromised mechanical sensitivity.

## Results

### The Cx43(M2) antibody enhances the opening of Cx43 HCs in osteocytes during mechanical unloading and loading in vivo.

**Figure 1:**
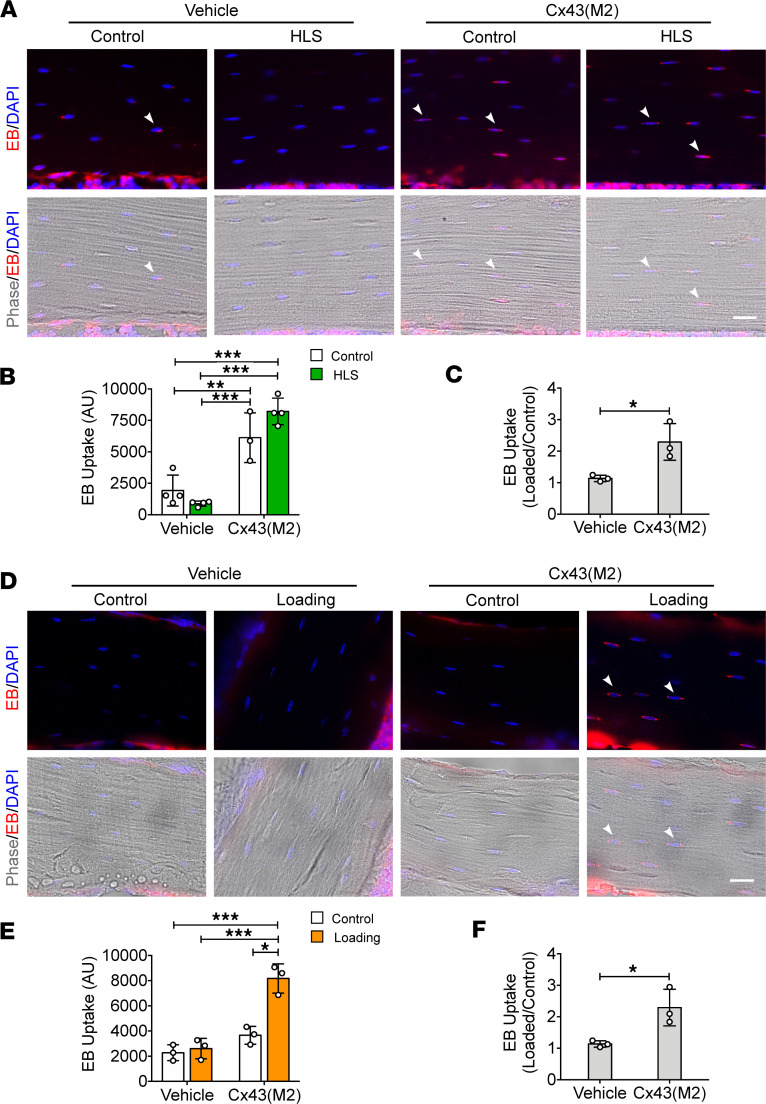
Cx43(M2) antibody enhances HC opening in osteocytes under both tibia loading and unloading in vivo. To assess the effects of Cx43(M2) on HCs in osteocytes, EB dye uptake was evaluated in 16-week-old male mice after 28-day HLS or in 22-month-old male mice after a single round tibia loading. (**A**) Representative fluorescence images of EB dye uptake in middiaphyseal cortical bone for both control and HLS unloaded tibias, in the absence or presence of Cx43(M2). White arrowheads indicate EB^+^ osteocytes. Scale bar: 20 μm. (**B** and **C**) Quantitation of EB fluorescence intensity and the ratio changes over control in osteocytes. *n* = 3–4 per group. (**D**) Representative fluorescence images of EB dye uptake in 37% diaphyseal cortical bone for both loaded and contralateral tibias, in the absence or presence of Cx43(M2). White arrowheads indicate EB^+^ osteocytes. Scale bar: 20 μm. (**E** and **F**) Quantitation of EB fluorescence intensity and the ratio changes over contralateral control in osteocytes. *n* = 3 per group. Data are presented as mean ± SD. *P < 0.05; **P < 0.01; ***P < 0.001. Statistical analysis was performed using the paired Student’s *t* test for loaded and contralateral tibias (**B** and **E**), unpaired Student’s *t* test for the ratio changes of EB dye uptake (**C** and **F**), and 2-way ANOVA with Tukey test for differences among groups (**B** and **E**).

To investigate the effect of monoclonal antibody Cx43(M2) on Cx43 HC activity in osteocytes, we performed the in situ Evans blue (EB) dye uptake assay, as previously described (18, 23, 24, 33). After a 7-day acclimatization period, 16-week-old WT mice were i.p. injected with 25 mg/kg Cx43(M2) or vehicle once a week. Mechanical unloading through HLS did not affect the basal dye uptake in osteocytes at the middiaphyseal bone region, whereas Cx43(M2) induced a strong EB uptake in both ground control and HLS tibias compared with vehicle-treated tibias (Figure 1, A and B). The fold change in HLS to the ground control indicated a significant increase in EB uptake by osteocytes in Cx43(M2)-treated mice but a decrease in vehicle-treated mice (Figure 1C). Our previous studies show that mechanical stimulation induces the opening of osteocytic Cx43 HCs in WT adult mice (23, 25). In contrast, loading did not increase EB uptake in osteocytes at the middiaphyseal region in 22-month-old aged mice in response to mechanical loading (Figure 1, D and E). Although Cx43(M2) did not increase EB uptake in osteocytes in contralateral control tibias, the increased dye uptake was observed with the combined treatment of Cx43(M2) and mechanical loading (Figure 1, D and E). EB fluorescence intensity in Cx43(M2)-loaded tibias was even greater than that of both vehicle-control and vehicle-loaded tibias (Figure 1E). The fold change determination indicated a significant increase in osteocyte EB uptake in Cx43(M2)-treated and mechanically loaded tibias (Figure 1F). Similarly, Cx43(M2) improved EB dye uptake under mechanical loading in the metaphyseal trabecular bone. Still, such an increase was not observed in vehicle-treated mice (Supplemental Figure 1; supplemental material available online with this article; https://doi.org/10.1172/jci.insight.177557DS1).

### Cx43(M2) prevents cortical bone loss caused by mechanical unloading.

The baseline body weight of mice was not different among the 4 experimental groups (Supplemental Figure 2A). Although Cx43(M2) caused a slight weight loss in ground control mice during the first 7 days of HLS, the weight was gradually regained (Supplemental Figure 2A). There was an obvious decrease in the body weight of mice subjected to unloading compared with ground control mice that were individually housed in a standard cage without tail suspension during the 4 weeks of HLS, but the unloading-induced decrease was not observed in Cx43(M2)-treated mice. On day 28, the unloading-induced loss of body weight was significantly less in Cx43(M2)-treated mice than in vehicle-treated mice (Supplemental Figure 2A).

**Figure 2:**
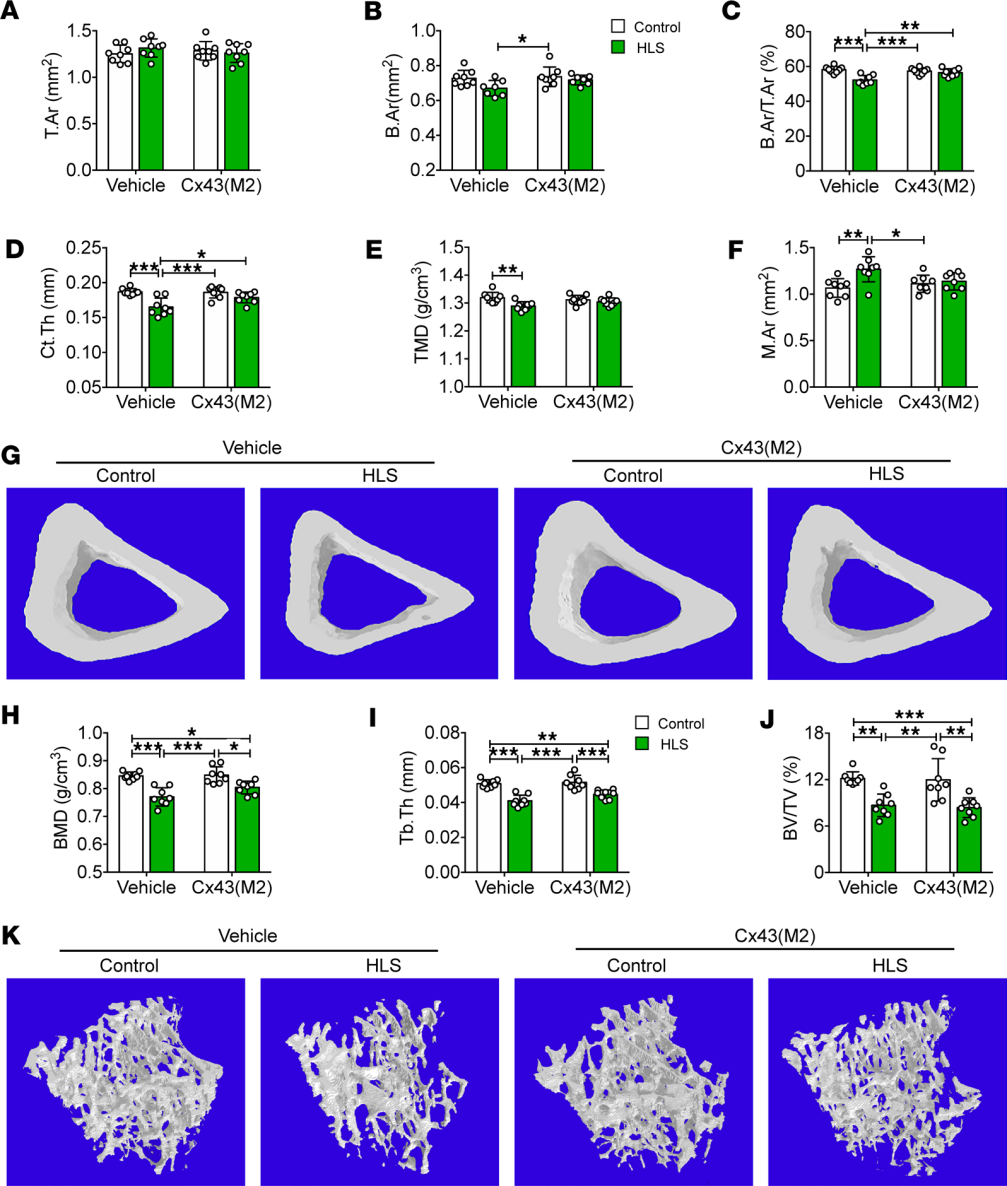
Enhanced Cx43 HC activity by Cx43(M2) prevents cortical bone loss caused by mechanical unloading. Sixteen-week-old male mice were subjected to a 28-day HLS to examine bone structure and mechanical properties. (**A**–**G**) μCT was used to assess structural parameters of middiaphyseal cortical bone: T.Ar (**A**), B.Ar (**B**), B.Ar/T.Ar (**C**), Ct.Th (**D**), TMD (**E**), and M.Ar (**F**). (**G**) Representative 3D models of tibial cortical bone in vehicle- and Cx43(M2)-treated mice. *n* = 8 per group. (**H**–**K**) μCT was used to assess structural parameters of trabecular bone: BMD (**H**), Tb.Th (**I**), and BV/TV in vehicle- and Cx43(M2)-treated mice (**J**). *n* = 7–8 per group. (**K**) Representative 3D models of the metaphyseal trabecular bone of vehicle- and Cx43(M2)-treated mice. Data are presented as mean ± SD. *P < 0.05; **P < 0.01; ***P < 0.001. Statistical analysis was performed using 2-way ANOVA with Tukey test for differences among groups (**A**–**F** and **H**–**J**).

The μCT analysis of the tibial cortical bone showed that 4 weeks of mechanical unloading via HLS did not alter the total cross-sectional area (T.Ar) and bone area (B.Ar) (Figure 2, A and B) but compromised the quality and structure of cortical bone in vehicle-treated mice, as indicated by bone area ratio (B.Ar/T.Ar), cortical thickness (Ct.Th), and tissue mineral density (TMD), as well as enlarged bone marrow area (M.Ar) (Figure 2, C–F). In contrast, these parameters remained unchanged between Cx43(M2)-control and Cx43(M2)-HLS mice. The B.Ar, B.Ar/T.Ar, and Ct.Th was significantly lower in vehicle-HLS tibias compared with vehicle-control or Cx43(M2)-HLS tibias (Figure 2, C and D), which was likely by the expanded M.Ar (Figure 2F). Representative images of cortical bones are shown in Figure 2G. In contrast, 4-week HLS caused significant loss of metaphysis trabecular bone, as shown by decreased bone mineral density (BMD), trabecular thickness (Tb.Th), and bone volume fraction (BV/TV) (Figure 2, H–J) in both vehicle-treated and Cx43(M2)-treated mice. However, the Cx43(M2) antibody did not rescue the metaphysis trabecular bone loss during HLS. A significant decrease of BMD, Tb.Th, and BV/TV was observed between vehicle-HLS and Cx43(M2)-control tibias and between vehicle-control and Cx43(M2)-HLS tibias (Figure 2, H–J). Representative images of trabecular bone are shown in Figure 2K. Interestingly, 3-point bending testing of whole femurs showed that the reduced elastic modulus, ultimate force, and ultimate stress and stiffness caused by unloading were not reversed by Cx43(M2) antibodies (Supplemental Figure 3, A–D). Together, these results suggest that Cx43 HCs enhanced by Cx43(M2) prevent bone loss and microstructure changes in cortical bone during mechanical unloading.

### Enhanced Cx43 HC activity inhibited osteocyte apoptosis and osteoclast activity caused by mechanical unloading.

**Figure 3:**
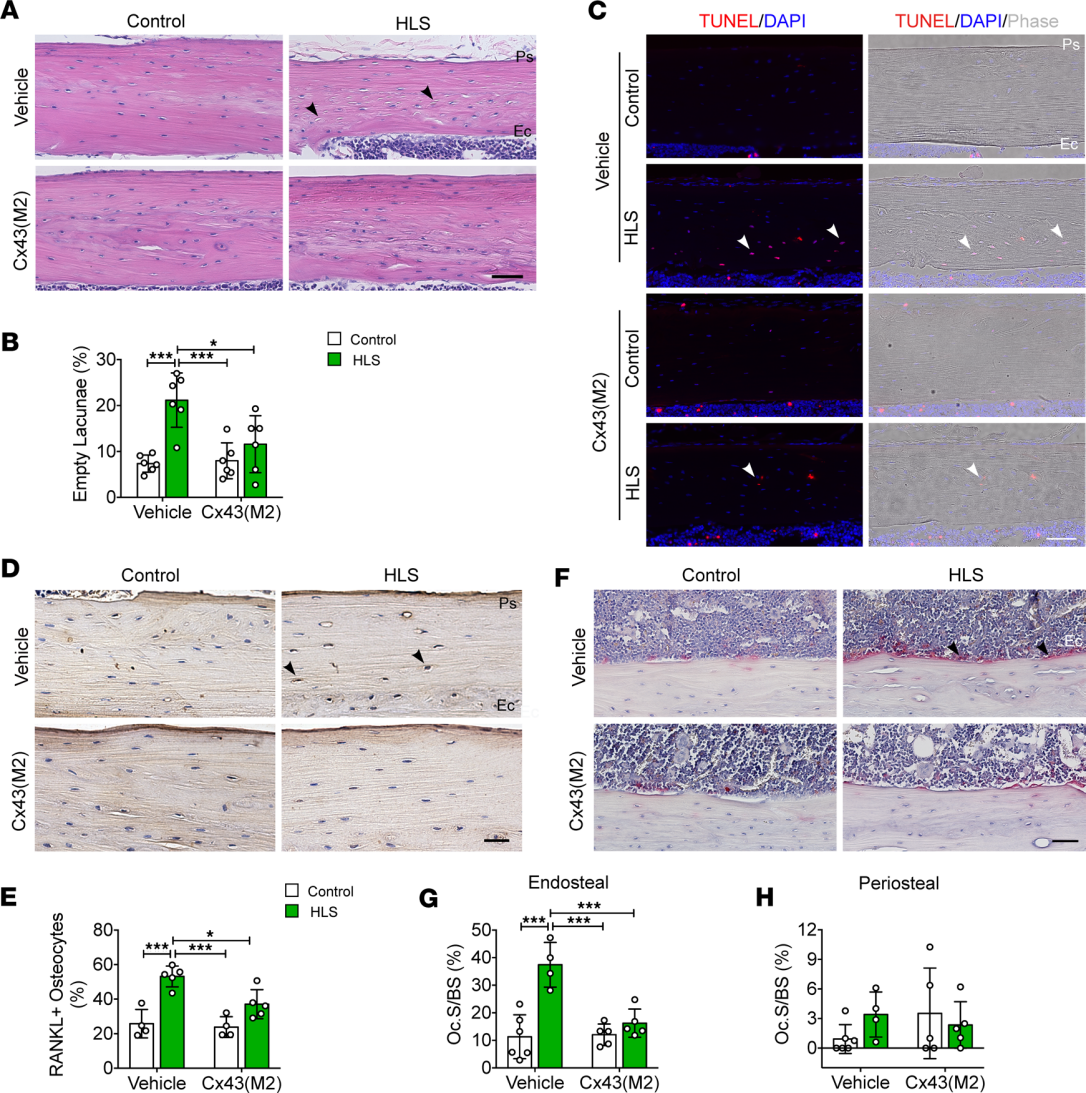
Enhanced Cx43 HC activity by Cx43(M2) inhibits osteocyte apoptosis and osteoclastogenesis in 16-week-old male tibias induced by 28-day mechanical unloading. (**A**) Representative images of H&E staining on middiaphyseal cortical bone for both control and HLS tibias of vehicle- and Cx43(M2)-treated mice. Black arrowheads indicate empty lacunae. *n* = 5–6 per group. Scale bar: 80 μm. (**B**) Quantification of empty lacunae in middiaphyseal cortical bone. (**C**) Representative images of TUNEL staining of apoptotic osteocytes in both control and HLS tibias of vehicle- and Cx43(M2)-treated mice. White arrowheads indicate TUNEL^+^ osteocytes. *n* = 4 per group. Scale bar: 120 μm. (**D** and **E**) Representative RANKL immunohistostaining and quantification of RANKL^+^ osteocytes in middiaphyseal cortical bone for both control and HLS tibias of vehicle- and Cx43(M2)-treated mice. Black arrowheads indicated RANKL^+^ osteocytes. *n* = 4 per group. Scale bar: 40 μm. (**F**) Representative images of TRAP^+^ osteoclasts (black arrows) on the endosteal surface for both control and HLS tibias of vehicle- and Cx43(M2)-treated mice. (**G** and **H**) Quantification of TRAP^+^ osteoclast surface per bone perimeter (Oc.S/BS) on endosteal surfaces (**G**) and periosteal surfaces of middiaphyseal cortical bone (**H**). *n* = 4–6 per group. Scale bar: 40 μm. Data are expressed as mean ± SD. *P < 0.05; ***P < 0.001. Statistical analysis was performed using 2-way ANOVA with Tukey test for differences among groups (**B**, **E**, **G**, and **H**). Ps, periosteal surface; Ec, endosteal surface.

H&E staining of tibial cortical bone revealed a higher number of empty lacunae close to the endosteal surface in vehicle-treated mice subjected to mechanical unloading via HLS. In contrast, Cx43(M2) inhibited the increase in empty lacunae by HLS, as shown by no significant difference of empty lacunae between Cx43(M2)-control and Cx43(M2)-HLS tibias, and the empty lacunae in Cx43(M2)-treated tibias were even lower than vehicle-HLS tibias (Figure 3, A and B). Apoptotic osteocytes were identified using TUNEL staining. In the middiaphyseal tibial bone, TUNEL^+^ osteocytes (red) (indicated by solid white arrows) were increased in vehicle-treated groups during mechanical unloading, but this increase was inhibited in Cx43(M2)-treated groups (Figure 3C). Interestingly, HLS caused a noticeable increase in TUNEL^+^ osteocytes close to the endosteal surface, whereas Cx43(M2) decreased osteocyte apoptosis in that region (Figure 3C). Osteocyte apoptosis induced by HLS is known to trigger osteocyte RANKL expression and osteoclast-mediated resorption (34, 35). IHC staining of tibial cortical bone showed a significant increase in RANKL^+^ osteocytes close to the endosteal surface due to mechanical unloading in vehicle-treated mice (Figure 3D). In contrast, the RANKL^+^ osteocytes did not significantly increase in Cx43(M2)-treated mice and were even lower than vehicle-HLS tibias (Figure 3, D and E). Tartrate-resistant acid phosphatase (TRAP) staining demonstrated an increase in osteoclast surfaces on the endosteum in response to mechanical unloading. In contrast, the Cx43(M2)-treated mice demonstrated effectively inhibited osteoclast activity and even showed lower osteoclasts compared with vehicle-HLS tibias (Figure 3, F and G). Osteoclasts on the endosteal cortical bone are depicted in Figure 3F. Notably, mechanical unloading did not affect periosteal osteoclast activity in either vehicle- or Cx4(M2)-treated mice (Figure 3H). These results illustrate that enhanced HC activity due to Cx43(M2) prevented unloading-induced osteocyte apoptosis and osteoclast resorption close to the endosteal surface.

### Enhanced Cx43 HC activity increased PGE_2_ release and suppressed sclerostin (SOST) expression in osteocytes during unloading.

**Figure 4:**
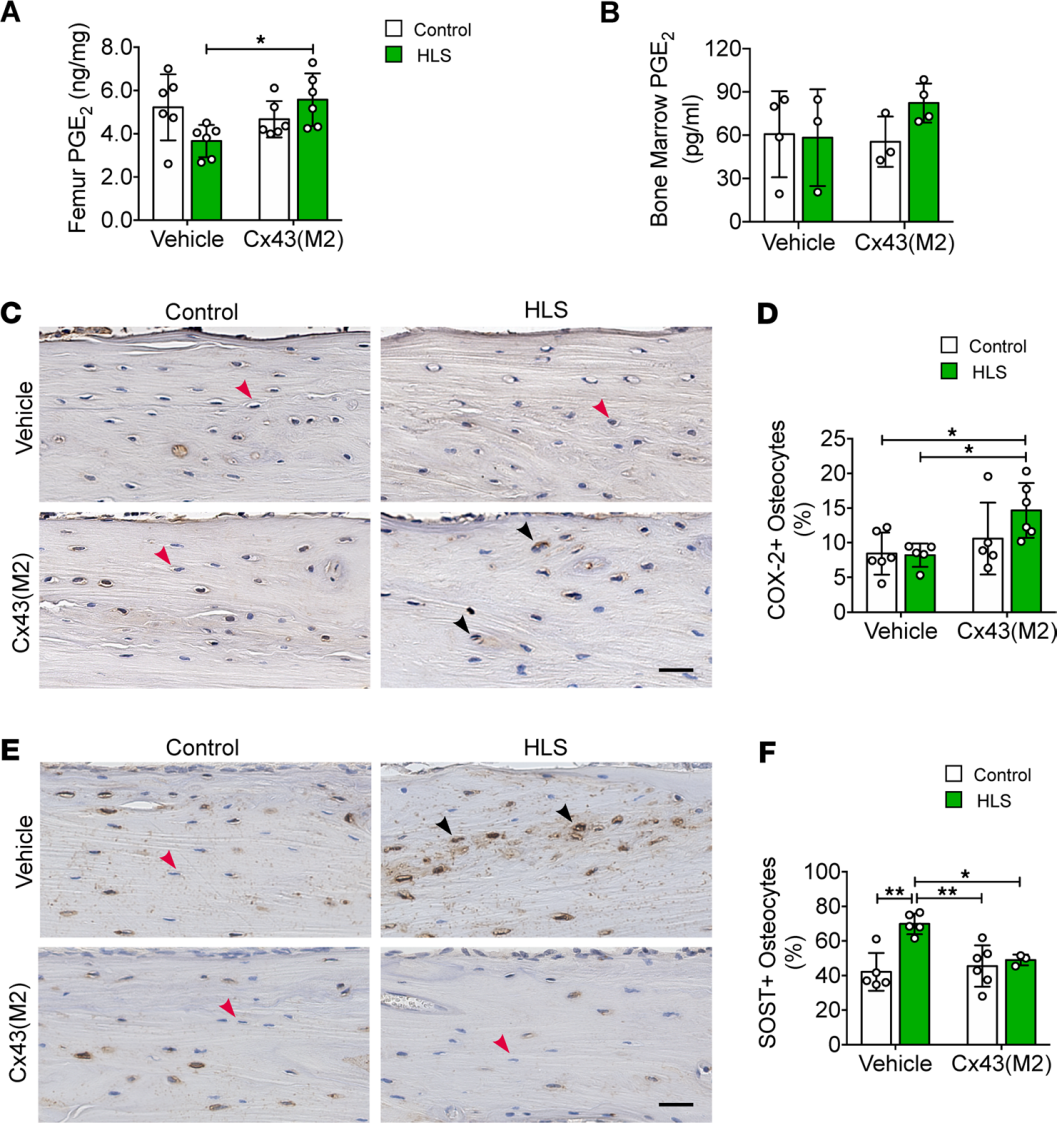
Cx43(M2) sustains PGE_2_ levels and prevents increased SOST expression in osteocytes caused by mechanical unloading. After a 4-week HLS in 16-week-old male mice, PGE_2_ levels and SOST were assessed. (**A** and **B**) ELISA analysis of PGE_2_ level in bone marrow–flushed femur diaphysis (**A**) and hindlimb bone marrow for both control and HLS tibias of vehicle- and Cx43(M2)-treated mice (**B**). (**C** and **D**) Representative COX-2 immunohistostaining and quantification of COX-2^+^ osteocytes (black arrows) in middiaphyseal cortical bone. Scale bar: 40 μm. *n* = 5–6 per group. (**E** and **F**) Representative SOST immunohistostaining and quantification of SOST^+^ osteocytes (black arrows) in middiaphyseal cortical bone. Scale bar: 40 μm. *n* = 5–6 per group. Black and red arrowheads indicate positive and negative osteocytes, respectively. Data are expressed as mean ± SD. *P < 0.05; **P < 0.01. Statistical analysis was performed using 2-way ANOVA with Tukey test for differences among groups (**A**, **B**, **D**, and **F**).

PGE_2_, released through Cx43 HCs, acts in an autocrine manner to reduce osteocyte apoptosis (21, 22). In this study, we measured the PGE_2_ levels in the femurs of both vehicle and Cx43(M2) groups after 2 weeks of unloading. Although there was no significant increase in PGE_2_ levels in femur bone between Cx43(M2)-control and Cx43(M2)-HLS mice, Cx43(M2)-HLS mice exhibited higher PGE_2_ levels than vehicle-HLS mice (Figure 4A). However, there was no significant change in the PGE_2_ levels in the hindlimb bone marrow (Figure 4B). Consistent with the PGE_2_ levels, IHC analysis revealed greater numbers of cyclooxygenase-2^+^ (COX-2, an inducible enzyme responsible for PGE_2_ synthesis) osteocytes in middiaphyseal tibial cortical bone of Cx43(M2)-HLS mice compared with both vehicle-HLS mice and vehicle-control mice (Figure 4, C and D). Osteocyte-derived SOST is a potent inhibitor of bone formation and osteogenesis (36), and previous studies have shown that PGE_2_ suppresses SOST expression in osteocytes (23, 24). Here, we investigated the effect of Cx43(M2) on HLS-induced SOST expression. Mechanical unloading increased the number of SOST^+^ osteocytes in vehicle-treated mice (Figure 4, E and F). In contrast, Cx43(M2) suppressed the increase in SOST expression in osteocytes caused by HLS. The SOST^+^ osteocytes were even lower in both Cx43(M2)-control and Cx43(M2)-HLS tibias compared with vehicle-HLS tibias. Additionally, dynamic histomorphometry showed that the bone formation rate per bone surface (BFR/BS) and mineral apposition rate (MAR) significantly decreased on the endosteal cortical surface in vehicle-treated mice during HLS (Supplemental Figure 4, A and B). Conversely, there were no changes in MAR, BFR/BS, or mineralizing surface per surface BS (MS/BS) in the Cx43(M2) groups (Supplemental Figure 4, A–C). Images of endosteal bone formation, indicated by double calcein labeling, are shown in Supplemental Figure 4D. Together, these results indicate that enhanced Cx43 HCs retained the ability to release PGE_2_ and suppressed the increase in SOST in osteocytes, correlating with osteoblast activity on the endosteal surface.

### Enhancing osteocytic Cx43 HCs with Cx43(M2) improves load-induced increase in trabecular and cortical microstructure in aged mice.

Next, we directly tested the hypothesis that the attenuated anabolic response to mechanical stimulation in aged bone was due to impaired Cx43 HC activity. The left tibias of 22-month-old vehicle control and Cx43(M2)-treated mice were mechanically loaded with a 9N compressive axial force, as illustrated in Supplemental Figure 5C. All mice showed evidence of good health throughout the loading experiment. A slight, temporary decline in body weight was observed during the first week of mechanical loading, and it stabilized by the second week (Supplemental Figure 5D). The Cx43(M2) treatment had a negligible effect on body weight (Supplemental Figure 5D).

**Figure 5:**
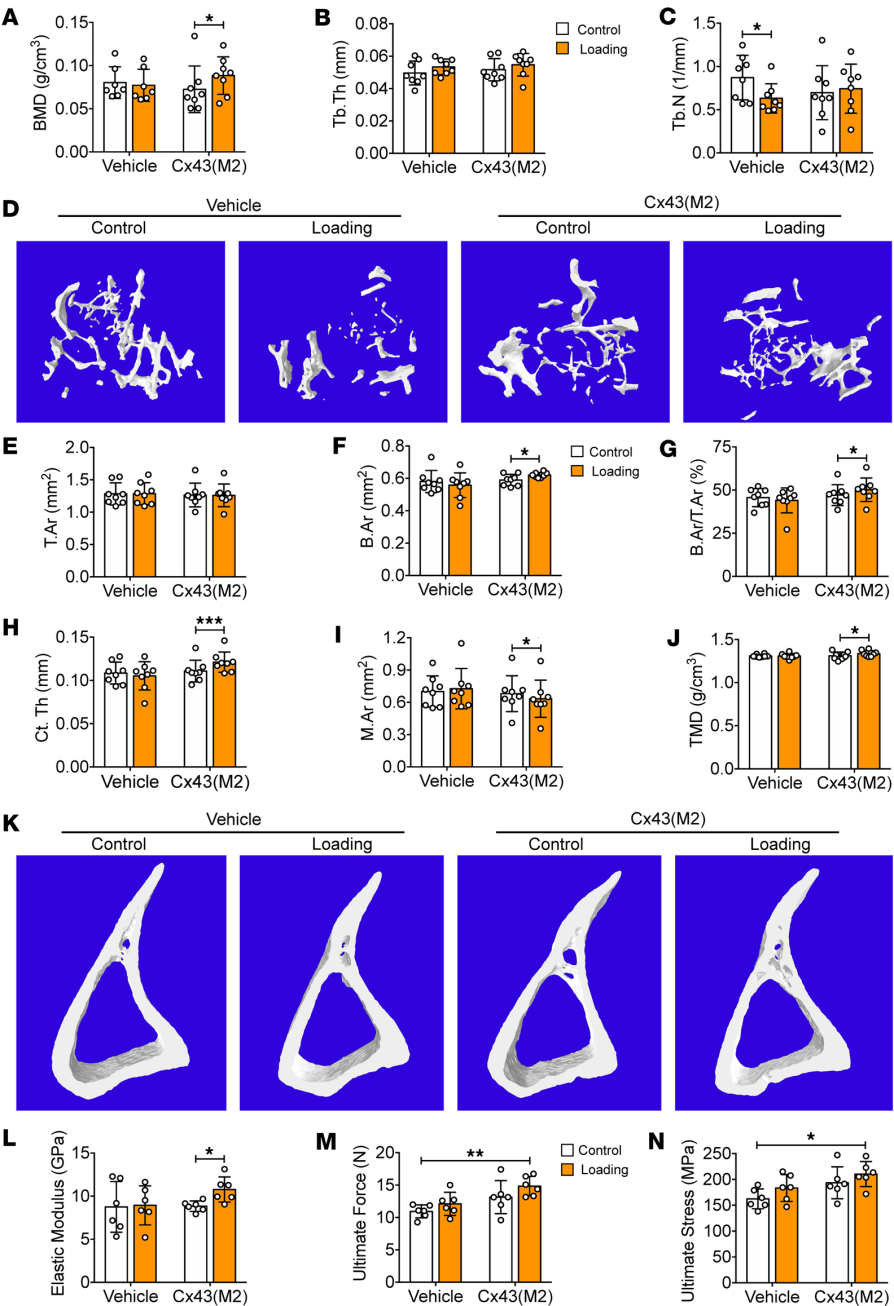
Cx43(M2) increases the anabolic effects of mechanical loading in aged mice. Bone structure and mechanical properties were analyzed in 22-month-old male mice subjected to loading 5 days/week for 2 weeks. (**A**–**C**) μCT was used to assess structural parameters of trabecular bone: BMD (**A**), Tb.Th (**B**), and Tb.N (**C**) of vehicle and Cx43(M2)-treated mice. *n* = 8 per group. (**D**) Representative 3D models of the metaphyseal trabecular bone of vehicle and Cx43(M2)-treated mice. (**E**–**J**) μCT was used to assess structural parameters of cortical bone located 37% distal from the proximal end: T.Ar (**E**), B.Ar (**F**), B.Ar/T.Ar (**G**), Ct.Th (**H**), M.Ar (**I**), and TMD (**J**). (**K**) Representative 3D models of the cortical bone in vehicle- and Cx43(M2)-treated mice. *n* = 7 per group. (**L**–**N**) The 3-point bending assay was performed for tibial bone of vehicle- and Cx43(M2)-treated mice: elastic modulus (**L**), ultimate force (**M**), and ultimate stress (**N**). *n* = 6 per group. Data are expressed as mean ± SD. *P < 0.05; **P < 0.01; ***P < 0.001. Statistical analysis was performed using the paired Student’s *t* test for loaded and contralateral tibias (**A**–**C**, **E**–**J**, and **L**–**N**), and 2-way ANOVA with Tukey test for differences among groups (**A**–**C**, **E**–**J**, and **L**–**N**).

μCT analysis on the metaphyseal region showed that, compared with the contralateral control tibias, 2-week tibial loading did not increase BMD or Tb.Th (Figure 5, A and B) but resulted in decreased trabecular number (Tb.N) (Figure 5C) in vehicle-aged mice. In contrast, Cx43(M2) treatment significantly increased BMD compared with the contralateral control tibias in response to mechanical loading. Representative images of trabecular bone are shown in Figure 5D.

We chose the diaphysis located 37% distal from the proximal end, where previous studies have shown the greatest osteogenic response to axial loading (37, 38), to analyze cortical bone morphology. Similar to the response in trabecular bone, cortical bone parameters of vehicle control mice, including T.Ar, B.Ar, B.Ar/T.Ar, Ct.Th, M.Ar, and TMD, were unresponsive to tibial loading (Figure 5, E–J). In comparison, Cx43(M2) treatment increased cortical B.Ar (Figure 5F) and Ct.Th (Figure 5H) by decreasing M.Ar (Figure 5I) without changing the T.Ar (Figure 5E) in loaded tibias, leading to significant increases in B.Ar/T.Ar and TMD (Figure 5, G and J) compared with the contralateral nonloaded control tibias. Representative images of cortical bone are shown in Figure 5K. Mechanical property analysis revealed a significantly increased elastic modulus over the contralateral controls in Cx43(M2)-treated mice, while such an increase was not observed in vehicle-treated mice (Figure 5L). Moreover, Cx43(M2)-loaded tibias exhibited significantly greater ultimate force (Figure 5M) and ultimate stress (Figure 5N) compared with vehicle control tibias. There was an increasing trend in bone stiffness in Cx43(M2)-treated mice during mechanical loading (Supplemental Figure 3E). In summary, enhanced Cx43 HC activity increased the anabolic response to loading in both aged trabecular and cortical bone.

To assess whether the Cx43(M2) antibody exerts any effect on the response to mechanical loading in young adult mice, 16-week-old male mice were subjected to tibial loading. μCT analysis of the diaphysis located 37% distal from the proximal end indicated that the endosteal bone formation response declines with age. Unlike aged mice, the cortical bone in both Cx43(M2)- and vehicle-treated young adult mice responded similarly to mechanical loading. Parameters such as B.Ar, B.Ar/T.Ar, and Ct.Th increased in the loaded group compared with contralateral, unloaded tibias. The Cx43(M2) antibody did not exert additional effects on cortical bone during mechanical stimulation in young adult mice (Supplemental Figure 6). Furthermore, bone histomorphometry analysis confirmed the μCT data, showing that mechanical loading significantly increased bone formation and MARs on both endosteal and periosteal BSs. However, the Cx43(M2) antibody did not provide further enhancement (Supplemental Figure 7), as shown by the significant difference between vehicle-loaded and Cx43(M2)-control tibias or between vehicle-control and Cx43(M2)-loaded tibias. Thus, the Cx43(M2) has no additional effect on tibial bone during mechanical stimulation in young adult mice.

### Enhancing Cx43 HCs in osteocytes increases endosteal anabolism in response to mechanical loading.

**Figure 6:**
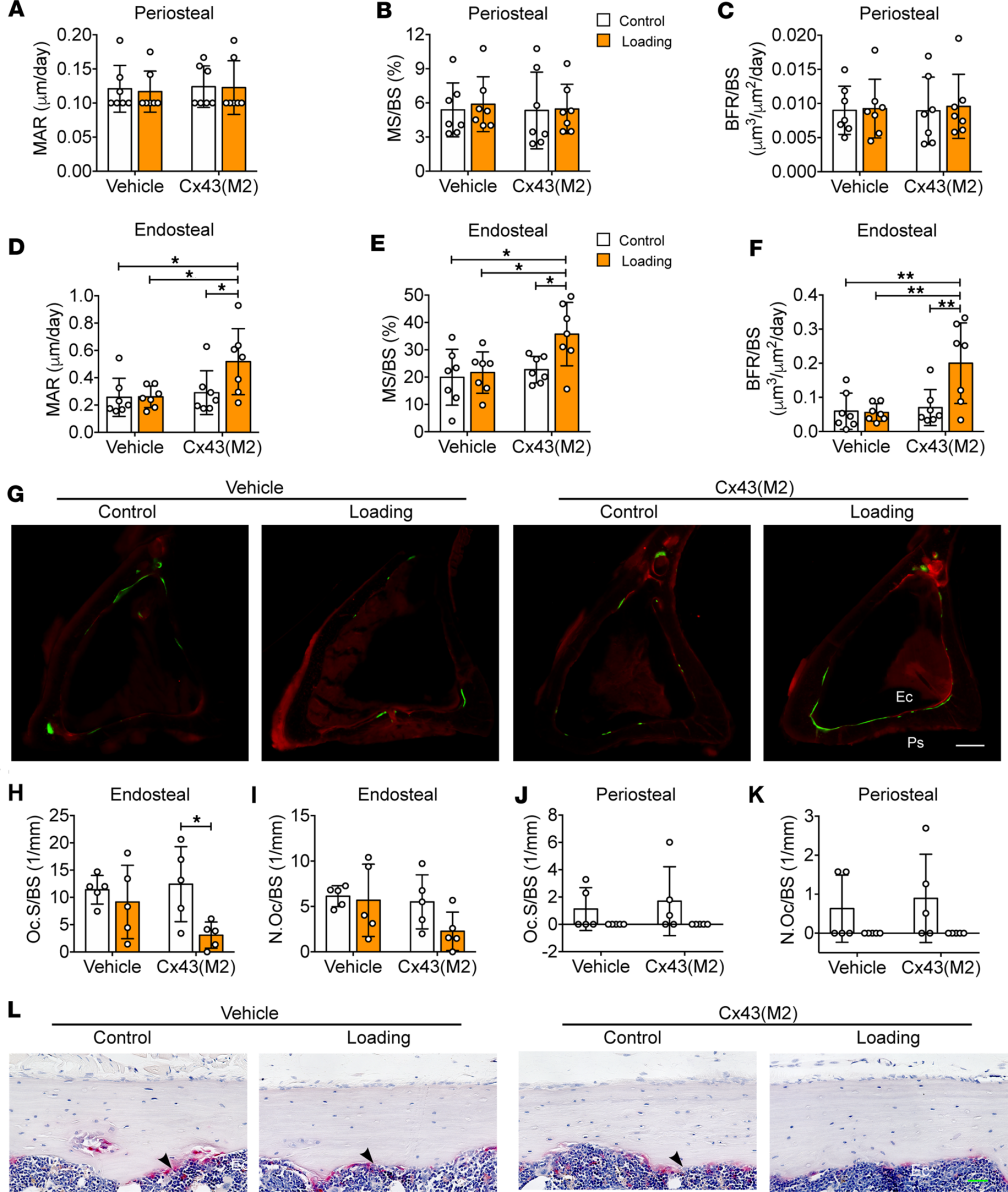
Enhanced Cx43 HC activity improves load-induced endosteal osteogenesis in aged mice. After 5 days per week of loading for 2 weeks, bone histomorphometry analyses were performed on tibias within cortical bone located 37% distal from the proximal end in 22-month-old vehicle- and Cx43(M2)-treated mice. (**A**–**F**) MAR (**A** and **D**), MS/BS (**B** and **E**), and BFR/BS (**C** and **F**) were assessed along periosteal (**A**–**C**) and endosteal (**D**–**F**) surfaces of all tibias. *n* = 7 per group. (**G**) Representative images of calcein (green) and alizarin (red) double labeling at the 37% diaphysis for all groups. Scale bar: 200 μm. (**H**–**K**) Quantification of TRAP^+^ osteoclast surface per bone perimeter (Oc.S/BS) and osteoclast number per bone perimeter (N.Oc/BS) on endosteal (**H** and **I**) and periosteal (**J** and **K**) surfaces of 37% diaphysis. *n* = 5 per group. (**L**) Representative images of TRAP^+^ osteoclasts (black arrowheads) on cortical bone located 37% distal from the proximal end for both loaded and contralateral tibias of vehicle- and Cx43(M2)-treated mice. Scale bar: 40 μm. Data are expressed as mean ± SD. *P < 0.05; **P < 0.01. Statistical analysis was performed using the paired Student’s *t* test for loaded and contralateral tibias (**A**–**F** and **H**–**K**), and 2-way ANOVA with Tukey test for differences among groups (**A**–**F** and **H**–**K**). Ps, periosteal surface; Ec, endosteal surface.

The reduced M.Ar in Cx43(M2)-loaded tibias (Figure 5I) suggested changes in the activities of osteoblasts and osteoclasts on the endosteal surface. Consequently, histomorphometry analysis of the diaphysis located 37% distal from the proximal end was performed. Dynamic histomorphometry indicated that mechanical loading did not affect either endosteal or periosteal bone formation in vehicle-aged mice. Cx43(M2) did not alter periosteal MAR, MS/BS, or BFR/BS (Figure 6, A–C) but improved the MAR, MS/BS, and BFR/BS response to loading compared with the nonloaded control tibias (Figure 6, D–F). Notably, these bone formation parameters in Cx43(M2)-loaded tibias were even greater than that of both vehicle-control and vehicle-loaded tibias. Representative images are shown in Figure 6G. Mechanical loading led to a significant decrease in the osteoclast surface on the endosteal surface compared with contralateral nonloaded controls in Cx43(M2)-treated mice, but this decrease was not observed in vehicle-treated mice (Figure 6, H and I). A similar response was also found on the trabecular surface (Supplemental Figure 8, A–C). In contrast, mechanical loading did not affect periosteal osteoclast activity in either vehicle- or Cx43(M2)-treated tibias (Figure 6, J and K). Representative images of TRAP^+^ osteoclasts on the endosteal surface are shown in Figure 6L. Thus, enhanced Cx43 HCs by Cx43(M2) increased bone formation and decreased osteoclast resorption on the endosteal surface caused by mechanical loading, resulting in a decrease in bone marrow area (Figure 5I).

### Cx43(M2) enhances loading-induced PGE_2_ release and suppresses SOST expression.

**Figure 7:**
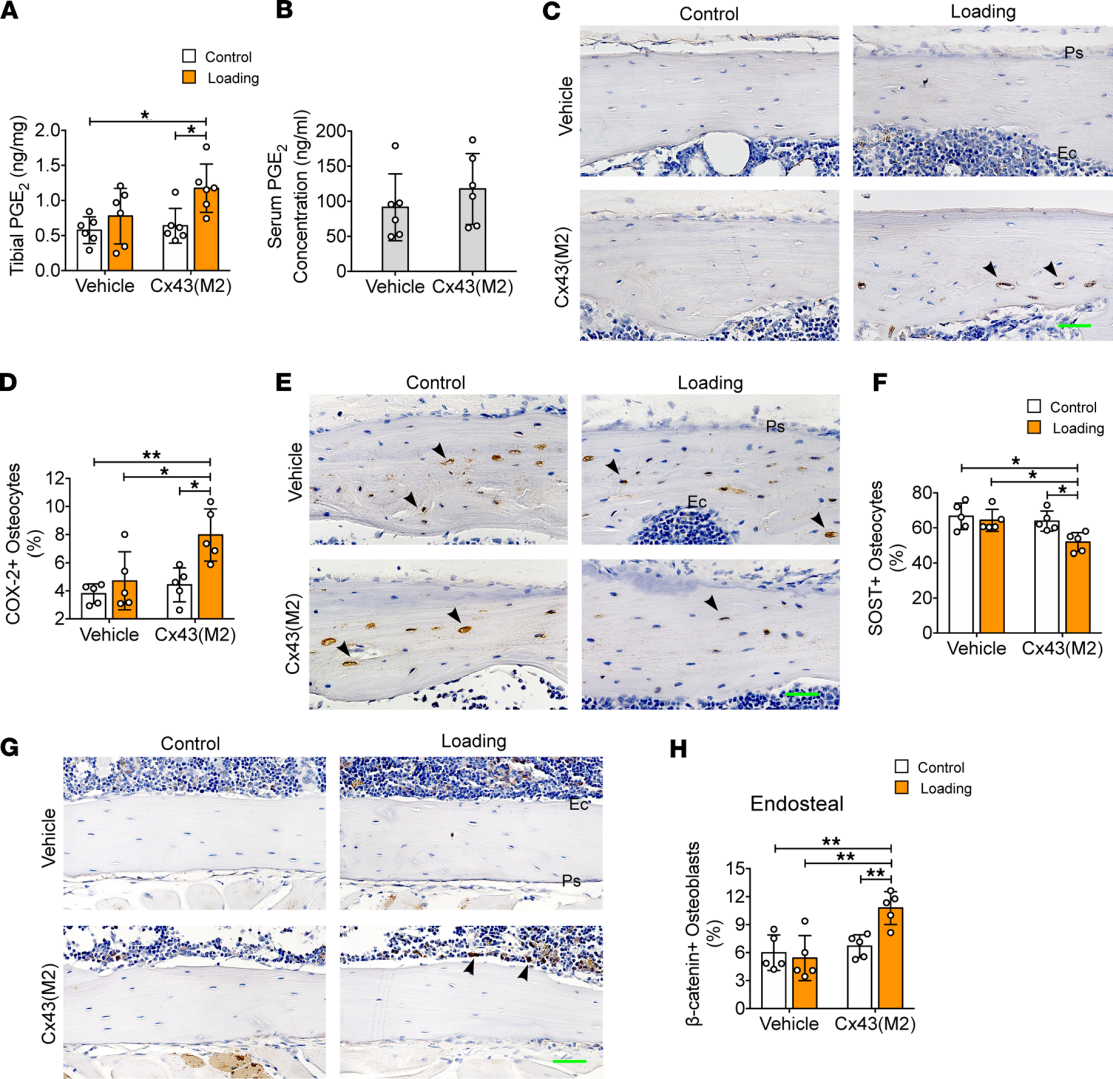
Enhanced activity of Cx43 HCs improves load-induced PGE_2_ secretion and decreases SOST expression in osteocytes of 22-month-old mice. (**A** and **B**) ELISA analysis of PGE_2_ level in bone marrow–flushed tibial diaphysis (**A**) and serum after 5 days of mechanical loading (**B**). *n* = 6 per group. IHC was performed on diaphyseal 37% cortical bone after 5 days/week loading for 2 weeks. (**C** and **D**) Representative COX-2 immunohistostaining and quantification of COX-2^+^ osteocytes in diaphyseal 37% cortical bone. Scale bar: 40 μm. *n* = 5 per group. (**E** and **F**) Representative SOST immunohistostaining and quantification of SOST^+^ osteocytes in diaphyseal 37% cortical bone. Scale bar: 40 μm. *n* = 5 per group. (**G** and **H**) Representative β-catenin immunohistostaining and quantification of β-catenin^+^ osteoblasts on the endosteal surface of diaphyseal 37% cortical bone. Scale bar: 50 μm. *n* = 5 per group. Black arrowheads indicate positive osteocytes. Data are expressed as mean ± SD. *P < 0.05; **P < 0.01. Statistical analysis was performed using the paired Student’s *t* test for loaded and contralateral tibias (**A**, **D**, **F**, and **H**), unpaired Student’s *t* test for the serum PGE_2_ level (**B**), and 2-way ANOVA with Tukey test for differences among groups (**A**, **D**, **F**, and **H**). Ps, periosteal surface; Ec, endosteal surface.

A significant increase in the PGE_2_ level was observed in loaded tibias compared with contralateral nonloaded control tibias in Cx43(M2)-treated mice. The PGE_2_ level in Cx43(M2)-loaded tibias was also greater than vehicle-control tibias (Figure 7A). Similar to unloading, we measured PGE_2_ earlier (5 days after loading) than μCT measurement after 2 weeks of loading because PGE_2_ is an early responsive factor to mechanical stimulation, and changes in bone structure are regulated by PGE_2_. The serum PGE_2_ level was similar between vehicle- and Cx43(M2)-treated mice (Figure 7B). Consistently, Cx43(M2) significantly increased COX-2 expression in the 37% diaphysis close to the endosteal region in loaded tibias compared with contralateral nonloaded controls, whereas the increased COX-2 expression was not detected in vehicle-treated mice (Figure 7, C and D). Moreover, the COX-2^+^ osteocytes in Cx43(M2)-loaded tibias were even more prevalent than that of vehicle-treated tibias (Figure 7D). We previously reported that mechanical loading downregulated the Wnt pathway antagonist, SOST, in osteocytes of 16-week-old WT mice (23, 24). Here, we found reduced SOST^+^ osteocytes in Cx43(M2)-treated tibias compared with contralateral nonloaded controls in the 37% diaphysis region and close to the endosteal region (Figure 7, E and F). In contrast, the SOST^+^ osteocytes were not reduced in vehicle-treated mice and were even more prevalent than in vehicle-treated tibias (Figure 7, D–F). Activation of β-catenin signaling in osteoblasts has been documented after 2-week mechanical loading in 16-week-old WT mice (23, 24); thus, we assessed the number of β-catenin^+^ osteoblasts in the diaphysis located 37% distal from the proximal end. Tibial loading upregulated β-catenin^+^ osteoblasts on the endosteal surface compared with the nonloaded contralateral controls in Cx43(M2)-treated mice. In contrast, the β-catenin^+^ osteoblasts did not increase in vehicle-treated mice, and their quantities were even lower than those of Cx43(M2)-loaded tibias (Figure 7, G and H). These results suggest that enhancing HCs by Cx43(M2) in osteocytes improves the anabolic responsiveness to mechanical loading in aged mice by upregulating the release of PGE_2_ and subsequently suppressing SOST in osteocytes. This suppression is correlated with increased levels of stable, unphosphorylated β-catenin and osteoblast activity on the endosteal surface.

## Discussion

**Figure 8:**
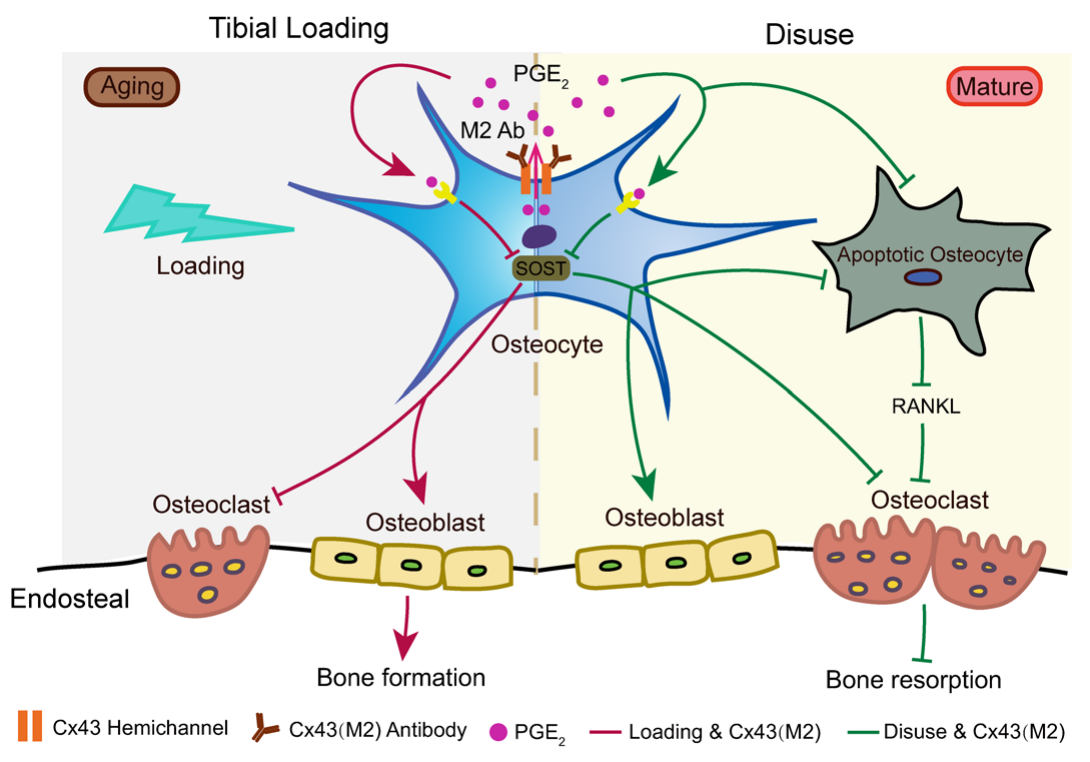
Model illustrating the effect of enhanced HCs via the Cx43(M2) antibody on osteocyte responses to mechanical loading in disused and aged bones. Enhanced Cx43 HC activity releases PGE_2_ (9), leading to a reduction of SOST expression in osteocytes during both mechanical loading and unloading. Aging is associated with a decline in osteocytic Cx43 levels and reduced responsiveness of Cx43 HCs to mechanical loading. Activation of osteocytic Cx43 HCs using the Cx43(M2) antibody increases PGE_2_ levels and suppresses SOST expression, resulting in increased endosteal osteoblast activity and bone formation, while simultaneously decreasing endosteal osteoclast activity in aged mice under mechanical loading conditions. During periods of disuse, apoptotic osteocytes release higher levels of RANKL, which induces osteoclast differentiation and recruitment. Activating HCs with the Cx43(M2) antibody raises extracellular PGE_2_ levels. This increase in PGE_2_ suppresses SOST, thereby enhancing endosteal bone formation and preventing osteocyte apoptosis and RANKL expression in osteocytes. Consequently, reduced endosteal osteoclast activity leads to decreased bone resorption.

Reduced sensitivity to mechanical stimulation associated with aging and disuse contribute to bone loss and osteoporosis. In this study, we demonstrated that increased activity of Cx43 HCs by a monoclonal antibody Cx43(M2) greatly mitigates the unloading-induced cortical bone loss in mature bone and reverses the unresponsiveness of aged bone to the anabolic functions of mechanical loading. The improved anabolic responses are associated with increased release of PGE_2_ from osteocytes (Figure 8).

In our previous studies using transgenic mouse models expressing dominant negative Cx43 mutants, we observed that impaired Cx43 HCs in osteocytes led to an increase in endosteal osteoclasts and bone loss during mechanical unloading (13), suggesting that Cx43 HCs play a protective role against mechanical unloading. Consistent with these findings, our study demonstrates that activation of Cx43 HCs by a Cx43(M2) protects against cortical bone loss caused by mechanical unloading. This protection was achieved by inhibiting increased osteoclast activity and preventing a decrease in bone formation. Interestingly, previous studies have shown that Cx43 cKO in osteoblasts/osteocytes preserves trabecular and cortical bone loss and attenuates osteoclast activity on the endosteal surface during unloading (14–16). A major difference between Cx43 cKO and transgenic mice expressing dominant negative Cx43 mutants is that Cx43 deficiency affects not only Cx43 channels but also other channel-independent functions.

Factors such as PGE_2_, released by Cx43 HCs activated through mechanical loading or the Cx43(M2) antibody, are the underlying mechanisms modulating bone formation and remodeling. Previous studies have identified PGE_2_ as a crucial anabolic factor mediating the effects of mechanical loading on bone (23, 24). Cx43 HCs act as the primary conduit for PGE_2_ release, activating EP2/EP4 receptors to promote bone formation and inhibit bone resorption (10, 25). Our recent study also showed that impairment of HCs in transgenic mice leads to reduced PGE_2_ production but that this effect can be rescued by PGE_2_ administration (24). In this study, we observed that Cx43(M2) treatment, similar to mechanical stimulation, increases extracellular PGE_2_ levels.

Previous research indicates that PGE_2_ released from osteocytes through Cx43 HCs suppresses SOST expression in osteocytes during mechanical loading (23, 24), and PGE_2_ treatment can suppress increased SOST expression in osteocytes caused by simulated microgravity (39). Our study found that Cx43(M2) inhibits the upregulation of SOST expression in osteocytes induced by unloading in mice. SOST is known to be associated with cell apoptosis (40). Previous studies have shown that unloading-induced upregulation of SOST expression induces osteocyte apoptosis by antagonizing the Wnt/β-catenin signaling pathway, while SOST deficiency attenuates osteocyte apoptosis in vivo (36). In addition to inhibiting SOST, PGE_2_ acts in an autocrine manner through EP2/4 receptors to block glucocorticoid-induced osteocyte apoptosis by increasing β-catenin level (21). This evidence suggests that PGE_2_ release through Cx43(M2)-enhanced Cx43 HCs rescues osteocyte apoptosis either directly or by suppressing SOST expression during mechanical unloading.

Cx43(M2) treatment markedly inhibited TRAP^+^ osteoclasts on the endosteal surface, which was associated with decreased osteocyte apoptosis close to the endosteal surface during mechanical unloading. These findings align with previous studies, which found that the highest levels of osteocyte apoptosis in cortical bone subjected to HLS were closest to the endosteal surface, resulting in RANKL^+^ osteocytes (35). In addition to osteocyte apoptosis, SOST is implicated in unloading-induced osteoclastogenesis and bone loss (36). SOST can directly increase RANKL levels from osteocytes, regulating osteoclast activity by inhibiting β-catenin in osteoclasts (41, 42). Conversely, SOST deficiency has been shown to resist bone resorption during mechanical unloading (36). Clinically, the antiosteoporosis drug romosozumab, which antagonizes SOST, has demonstrated antiresorptive effects in postmenopausal women with osteoporosis (43). Our study corroborates these findings, showing a suppressed increase of SOST^+^ osteocytes and reduced bone resorption with Cx43(M2) treatment after mechanical unloading. Furthermore, SOST not only promotes osteoclast formation but also affects osteoblasts, inhibiting bone formation (44). Previous studies have shown that mechanical unloading reduces bone formation on the endosteal surface (15, 45). Similarly, we observed a profound suppression of endosteal bone formation after mechanical unloading. Cx43(M2) completely ameliorated this unloading-induced effect, preserving bone formation. These findings align with the observation in SOST-KO mice (36). Together, these observations support the idea that the opening of Cx43 HCs suppressed osteocyte apoptosis and SOST expression caused by mechanical unloading, leading to decreased osteoclastogenesis and bone loss.

Conversely, Cx43(M2) did not rescue the trabecular bone loss caused by mechanical unloading. It is speculated that trabecular and cortical bone have different sensitivities to mechanical unloading. Trabecular bone has been reported to be more sensitive than cortical bone to skeletal unloading (46, 47) in rodents. Clinical studies have also demonstrated that trabecular bone rather than cortical bone is lost with reduced physical activity levels in young men (48). Another contradiction is that although cortical bone loss was rescued by Cx43(M2), the whole bone’s mechanical properties still degraded after unloading. It is likely that mechanical properties are not only related to changes in bone structure but also to those in bone components, such as collagen and noncollagen proteins (49).

Similar to disuse-induced bone loss from a lack of mechanical stimulation, aged bones lose their responsiveness to mechanical loading. Upon tibial loading, we observed no increase of bone mass in either cortical or trabecular bone in 22-month-old mice treated with the vehicle. In contrast, the same loading force (9N), which resulted in more strain in aged bone than in younger mice (27, 50), caused a marked increase in bone mass in 16-week-old WT mice, as reported in our previous study (24). Given that aged mice experience higher strain levels, the lack of responsiveness in periosteal bone formation is unlikely to be due to insufficient microstrain. Instead, it suggests that aging might affect the bone’s response to mechanical stimulation. It is noteworthy that the trabecular number decreased even further in the vehicle group during mechanical loading due to enhanced osteoclast resorption. A similar attenuated response was also observed in 20-month-old mice subjected to 11N loading (51). However, previous studies have shown that while mechanical loading induced endocortical bone formation in both young and aged mice, aged mice showed reduced bone formation across several parameters (26, 27). For instance, one study (27) calculated endosteal bone formation by combining lamellar and woven bone values. Reduced bone formation with aging was evident in several parameters, with a greater increase in endosteal BFR/BS^+^ and MAR^+^ in 5-month-old mice compared with 22-month-old mice in the antero-medial microstrain (1,200 με) group. Another study (26) showed a significant increase in mineralizing surface in loaded tibias of 5-month-old mice but no loading effect in 22-month-old mice. Here, our 22-month-old mice showed no response to antero-medial microstrain more than 1,200 με. We used male mice, while the other study used female mice. A previous study showed different mechanical responses between sexes. Estrogens have effects on endosteal bone apposition in females (52), which may explain the variation in woven bone formation on the endosteal surface.

It is plausible that the decreased Cx43 expression in osteocytes of aged mice (13) inhibited the function of mechanosensitive Cx43 HCs. Indeed, our recent studies show that inhibiting Cx43 HCs increases osteoclast activity and resorption in trabecular bone during mechanical loading in 16-week-old mice (24). Consistently, a similar load-induced increase in trabecular osteoclasts was also found in ovariectomized mice (53) that had reduced Cx43 HC function in osteocytes (54). In contrast, Cx43(M2) rescued the anabolic unresponsiveness of trabecular bone to tibial loading, as evidenced here by increased BMD and decreased osteoclast activity. These observations indicate enhanced Cx43 HC activity increases mechanical sensitivity and anabolic response in aged trabecular bone.

Previous studies have also shown a diminished endosteal bone formation response to mechanical loading in old mice of the same age (26, 55). Similarly, deletion of Cx43 from osteoblasts and osteocytes has also exhibited an attenuated increase (30, 31) or an even a greater decrease in endosteal bone formation during tibial loading (56, 57), suggesting that reduced Cx43 expression in osteocytes (13) affects the function of Cx43 HCs and, subsequently, the cortical osteogenic response to mechanical loading. In line with this observation, our previous studies have shown that inhibiting osteocytic Cx43 HCs decreases osteoblast-to-osteocyte differentiation in vitro (58) and suppresses the anabolic bone responses to mechanical loading on the endosteal surface in vivo (23, 24). Here, we showed that enhanced Cx43 HCs by Cx43(M2) significantly increased bone formation on the endosteal surface in aged mice. We observed a decrease in endosteal osteoclasts in Cx43(M2)-treated mice during mechanical loading. It is possible that factors (i.e., PGE_2_) released through activated HCs by Cx43(M2) may have marked negative effects on osteoclasts due to their predominant presence on the endosteal surface. Indeed, we found that the Cx43(M2) antibody promoted COX-2 expression in osteocytes near the endosteal surface. These findings align with our previous studies, where inhibition of Cx43 HCs in osteocytes promoted osteoclastogenesis in vitro (58) and accelerated bone resorption in vivo during mechanical stimulation (24). Notably, decreased SOST^+^ osteocytes were observed in the endosteal region but not in the periosteal region in the Cx43(M2) group. This may explain why β-catenin only increased on the endosteal surface in Cx43(M2)-loaded tibias. Consequently, activation of osteocytic Cx43 HCs by the combined treatment of Cx43(M2) and mechanical loading in aged bone promotes osteoblast recruitment and differentiation on the endosteal surface during axial compression loading. As a result, the reduced bone marrow cavity led to an increase in B.Ar, Ct.Th, and BMD and improved mechanical properties.

Cx43 HC opening induced by FFSS mediates the release of the bone anabolic factor PGE_2_ (10, 20), which is synthesized by osteocytes (59). The suppressed PGE_2_ level was also observed in our transgenic mouse models expressing dominant negative Cx43 mutants that inhibit Cx43 HCs (24) and in 8 kb *Dmp1-Cre;* Cx43 cKO mice after tibial loading (56). In this study, we found that Cx43 HC–deficient aged mice had a suppressed PGE_2_ level in the tibia bone after mechanical loading. However, the combined treatment with Cx43(M2) and mechanical loading activated osteocytic Cx43 HCs, a response that could not be achieved by either intervention alone. This combined treatment increased the release of PGE_2_ in aged tibias. It is worth noting that Cx43(M2) promoted the distribution of osteocyte expression COX-2, an enzyme responsible for PGE_2_ synthesis, near the endosteal surface during mechanical loading. Thus, extracellular PGE_2_ is a crucial modulator in promoting endosteal bone formation (60, 61), whereas PGE_2_ deficiency attenuates endosteal bone anabolism during mechanical loading (62). Our previous in vivo study found that loading-induced release of PGE_2_ from Cx43 HCs suppressesd SOST expression in osteocytes, thereby improving β-catenin expression in osteoblasts and osteogenesis (23, 24). Here, we showed that SOST expression was not suppressed in vehicle-treated aged mice. In contrast, SOST expression decreased near the endosteal surface when Cx43 HCs were enhanced in aged mice. Correspondingly, increased unphosphorylated β-catenin levels and bone formation were observed. These results indicate that enhanced Cx43 HC activity locally improved the release of PGE_2_ from osteocytes in mechanically insensitive aged mice. This, in turn, reduced SOST in osteocytes and increased β-catenin signaling in osteoblasts, ultimately leading to enhanced endosteal bone formation.

Interestingly, periosteal bone formation remained unresponsive to mechanical loading in aged mice, and even enhanced Cx43 HCs by Cx43(M2) did not promote the periosteal anabolic response to mechanical loading. Previous studies indicate that the anabolic response of aged cortical bone is more active on the endosteal surface than on the periosteal surface (63, 64). Consistently, our recent studies on 16-week-old mice also demonstrate that Cx43 HCs exclusively function in the anabolic response of the endosteum to mechanical loading (24). Here, our results indicate that enhanced Cx43 HCs by Cx43(M2) increased PGE_2_ synthesis and decreased SOST expression close to the endosteal surface, suggesting that these local effects may contribute to anabolic response specifically on the endosteal surface, not on the periosteal surface. However, our previous studies did not detect any differences in antibody distribution between the endosteal and periosteal surfaces (32). Further investigation is needed to understand the underlying mechanisms fully.

Notably, it is still unclear why mechanical loading cannot cause obvious opening of HCs in aging bone. One possibility is the age-related expression of integrins. Indeed, integrin α5, which regulates the opening of Cx43 HCs, is expressed less in old human fibroblasts than in young cells (65). Less integrin α5 likely attenuates the efficiency of loading-induced Cx43 HC opening. In contrast, Cx43(M2) needs to be combined with mechanical loading to activate HC opening in osteocytes in aged mice. We speculate that this is related to the more active osteocytes (66) and higher Cx43 expression (28) in younger mice compared with aged mice. Indeed, the Cx43(M2) antibody had no additional effect on load-induced cortical osteogenesis in 16-week-old young adult mice. HC opening induced by mechanical loading in osteocytes is likely reaching optimal levels, and the addition of Cx43(M2) would make limited contributions to HC activities. Therefore, the combined treatment of mechanical loading and Cx43(M2) in young adult mice does not further improve cortical bone responses to mechanical loading.

Current osteoporosis drugs, despite their efficacy, often come with adverse effects that may limit their long-term safety. For instance, antiresorptive agents such as bisphosphonates and the monoclonal antibody to RANKL (i.e., denosumab) not only inhibit bone resorption but also reduce bone formation, increasing the risk of osteonecrosis of the jaw and atypical femoral fracture (67). The anabolic agents such as parathyroid hormone (PTH) (i.e., teriparatide) or the PTH-related peptide analog abaloparatide stimulate both bone formation and bone resorption due to PTH’s ability to increase RANK production (68). Recently, the US Food and Drug Administration (FDA) approved a neutralizing antibody to SOST, romosozumab. However, the risk of oversuppression of bone remodeling and associated serious cardiovascular adverse events requires attention. Therefore, there is a pressing need to improve the efficacy and safety of osteoporosis drugs. More importantly, almost all osteoporosis drugs are more efficient on trabecular bone than cortical bone (69), despite fractures occurring predominantly in cortical bone (70). Increasing cortical bone formation poses a major challenge for existing therapies. In this study, we demonstrated that Cx43(M2) treatment not only enhances trabecular but also cortical bone formation. The humanized version of Cx43(M2) antibody, targeting osteocyte Cx43, is currently under clinical trial for treating osteosarcoma (71). Our current findings suggest that Cx43(M2) could serve as a candidate for developing drugs to treat bone loss and osteoporosis in aging and disuse populations.

## Methods

### Sex as a biological variable.

Adult (16-week-old) and aged (22-month-old) male mice were used in this study. We examined male mice because this ruled out the effect of estrogen on bone phenotype.

### Experimental animals.

Animal procedures were approved by the UTHSCSA IACUC. Male C57BL/6 mice from The Jackson Laboratory were housed in the animal care facility with a 12-hour light/dark cycle and a room temperature of 25°C under pathogen-free conditions at the UTHSCSA Institutional Lab Animal Research facility. Mice were housed with no more than 5 mice in each cage before experiments and were provided standard rodent chow.

### Monoclonal antibody Cx43(M2) generation and treatment.

The monoclonal Cx43(M2) antibody was generated and characterized as detailed in our recent publication (32). Briefly, mice were immunized with a Cx43 extracellular domain peptide (50 μg) i.p. in complete Freund’s adjuvant and then boosted repeatedly with the peptide antigen formulated in incomplete Freund’s adjuvant. Spleens were harvested, and fusions were performed as previously described (72, 73). After functional characterization of the hybridoma clones, the Cx43(M2) clone was selected, and the antibody was purified by affinity chromatography using protein A resin, as reported previously (74).

For mechanical unloading, after a 7-day acclimatization period, 16-week-old WT mice were i.p. injected with 25 mg/kg Cx43(M2) or vehicle (phosphate-buffered saline [PBS], pH 7.4) once a week (Supplemental Figure 2B). For mechanical loading, randomly allocated 22-month-old WT mice were i.p. injected with 25 mg/kg Cx43(M2) or vehicle [PBS], pH 7.4) the day before tibial suspension. A second dose was administered the day before the start of loading in the second week (Supplemental Figure 5A). The dosage and frequency were determined based on our previous study (23), primarily aligning with the tibial loading protocol commonly used for 2 weeks to minimize callus bone formation while demonstrating anabolic effects (75).

### Mechanical unloading.

Mechanical unloading was achieved using the tail suspension (HLS) model modified from the method described by Ferreira and colleagues’ method (76). Briefly, 16-week-old vehicle-treated mice and Cx43(M2)-treated mice were divided into ground control and HLS groups. In the HLS groups, sterile copper was fixed to the tail of anesthetized mice (Supplemental Figure 2C). Two mice were housed per cage for all groups. The suspension height was adjusted using the copper wire to maintain the mice at about a 30° head-down tilt, while mice in the ground control group were allowed to move freely without tail-suspended. Body weight was measured every week.

### In vivo tibial loading.

During tibial loading, mice were under isoflurane inhalation. As described in our previous studies (23, 24), the effects of tibial loading were assessed in the left tibias of 22-month-old and 16-week-old mice treated with Cx43(M2) or vehicle. Briefly, the left tibia was placed in a customized apparatus with a continuous 0.5N static preload (Supplemental Figure 5C). For aged mice, a 9N force was then loaded on the left tibias with 600 cycles (5 minutes) at a 2 Hz frequency using a loading device (7528-10, Masterflex L/S). Previous studies reported that this force, compared with younger mice, led to more strain (about 1,650 με) onto the anterior-medial surface (77) but caused an attenuated bone formation response in old mice at diaphysis 37% distal from the proximal end (26, 55). A 2-week mechanical loading period was used for bone structure, bone formation, histology, and IHC assays, or 5 consecutive days for PGE_2_ determination (Supplemental Figure 5, A and B). Since PGE_2_ is an early responsive factor to mechanical stimulation and is involved in regulating bone structure, it was measured at earlier time points compared with the μCT measurements. The right tibias served as contralateral, nonloaded controls. Body weights were monitored every week during tibial loading. For young adult mice, tibias were loaded to the same strain (about 1,650 με) based on our previous strain measurements (24), and the same loading parameter was used for aged mice.

### μCT.

A μCT scanner (SkyScan 1172, Bruker Micro-CT) was used for bone structure scanning after 4-week mechanical unloading or 2 weeks of mechanical loading. For mechanical unloading, the tibial cortical VOI was positioned 70 slices (0.7 mm) distal to the proximal growth plate with an extension of 50 slices (0.5 mm) from the distal side. The middiaphyseal VOI was reported to respond to mechanical unloading (78). Cortical bone with a threshold of 106–256 was selected for analysis. The metaphyseal trabecular volume of interest (VOI) was positioned 45 slices (0.45 mm) distal to the proximal growth plate with an extension of 100 slices (1 mm) from the distal side, representing the secondary spongiosa. A grayscale threshold of 80–256 was used for the trabecular VOI analysis. For mechanical loading, the cortical VOI was positioned 50 slices (0.5 mm) centered at the diaphysis 37% distal from the end of the proximal side to analyze the response to mechanical loading. This VOI matches previously published studies examining dynamic loading of tibias (23, 26). The trabecular VOI is the same as the nonloaded ones. The structural morphometric properties of cortical and trabecular regions were analyzed using the CT-Analyser software (CTAn 1.18.8.0, Bruker Micro-CT).

### Bone mechanical and material property testing.

After 4 weeks of mechanical unloading or 2 weeks of mechanical loading, tibias were cleared of the soft tissue and subjected to a 3-point bending test along the medial-lateral direction in a micromechanical testing system (Mach-1 V500CST, Biomomentum), as described previously (23, 24). The loading parameters are an 8 mm span with a loading speed of 0.05 mm/sec and 200 Hz. Mechanical properties were calculated using the cross-sectional areas determined from μCT.

### Dynamic bone histomorphometry.

For the 2-week loading experiment, mice were i.p. injected with calcein (C0875, Sigma-Aldrich) 1 day before tibial loading, followed by an alizarin red injection (A5533, Sigma-Aldrich) 3 days before euthanization, as described previously (23, 24). For the 4-week unloading experiment, mice were i.p. injected with calcein 1 day before the start and end of mechanical unloading separately. Tibias were embedded in methyl methacrylate, and 80 μm–thick transversal sections of the middiaphyseal site were cut using a precision wafering saw (PICO 155, PACE Technologies). The sections were then sanded to a thickness of 80 μm using P1200 grit sandpaper on the grinder polisher (Phoenix 4000 Buehler). Fluorescent labels were imaged with a fluorescence microscope (BZ-X710, Keyence).

### Dye uptake in vivo study.

The in situ assessment of osteocytic HC activity in tibias was described previously (18, 24). Briefly, for the unloading experiment, 20 mg/mL EB dye was injected into the tail vein immediately after the completion of the 4-week mechanical unloading. After 4 hours, anesthetized mice underwent heart perfusion to fix the osteocytes in the tibias. For the loading experiment, 20 mg/mL EB dye was injected into the tail vein. After 10 minutes of loading and 40 minutes of rest, anesthetized mice underwent heart perfusion to fix the osteocytes in the tibias. Tibias were embedded in the sagittal orientation in optimum cutting temperature compound to cut 12 μm frozen sagittal sections. The nuclei were stained with DAPI. EB fluorescence intensity in osteocytes at the middiaphyseal site was quantified.

### PGE_2_ measurement.

The PGE_2_ level in the serum, femurs, tibias, and bone marrow from hindlimbs was quantified using the PGE_2_ ELISA kit (Cayman Chemical). Four hours after the completion of the 5-day tibial loading or 4-week mechanical unloading, serum, bone marrow, and tibial diaphysis without bone marrow and soft tissues were collected and stored at –80°C. Femurs and tibias were homogenized, and the PGE_2_ levels in all samples were quantified using the PGE_2_ ELISA kit (514010, Cayman Chemical) and calibrated to the total protein concentration determined by a BCA assay.

### Histology.

Four-week mechanical nonloaded or 2-week mechanical loaded tibias were decalcification and then embedded sagittally in paraffin blocks to obtain 5 μm–thick longitudinal sections. TRAP staining was performed to determine osteoclast activity, as described previously (79). Multinucleated (≥3 nuclei) TRAP^+^ osteoclasts were quantified on the endosteal and metaphyseal trabecular surface. H&E staining was used to quantify the number of empty lacunae. The *In Situ* Cell Death Detection Kit (121567929101, Roche) was utilized to detect apoptotic osteocytes in the middiaphyseal region, as previously reported (23, 79).

For IHC, we used an IHC kit (PK-4001 and PK-4005, Vectastain) and a DAB substrate kit (SK-4100, Burlingame). Briefly, after antigen retrieval, described previously (24), sections were probed with primary antibodies against RANKL (ab9957, 1:200, Abcam), COX-2 (12375-1-AP, 1:200, Proteintech), SOST (AF1589, 1:400, R&D Systems), and unphosphorylated β-catenin (ab16051, 1:200, Abcam), followed by counterstaining with hematoxylin. Images were captured using a microscope (BZ-X710, Keyence) and quantified using the NIH ImageJ software.

### Statistics.

Statistical analysis was conducted using GraphPad Prism Version 7 software. Variance homogeneity was evaluated using the Levene test, and normal distribution was determined by the Shapiro-Wilk test. All data are presented as mean ± SD. The paired 2-tailed Student’s *t* test was used to compare the contralateral and loaded tibias within each mouse. The unpaired 2-tailed Student’s *t* test was employed to compare the ratio changes of EB dye uptake and serum PGE_2_ levels between vehicle- and Cx43(M2)-treated mice. Two-way ANOVA with Tukey test was used for multiple-group comparisons. For all comparisons, *P* values of less than 0.05 were considered significant.

### Study approval.

All described animal protocols were reviewed and approved by the UTHSCSA IACUC, in accordance with policies dictated by the Office of Animal Welfare at the NIH, USA.

### Data availability.

Values for all data points in graphs are reported in the Supporting Data Values file and are available from the corresponding author upon request.

## Author contributions

DZ, CT, and JXJ conceived the idea and designed the experiments. DZ, CT, LZ, TG, and SG performed the experiments. DZ and CT analyzed the data and designed the figures. DZ wrote the first draft of the manuscript, and JXJ revised the manuscript. JXJ wrote the definitive manuscript. JXJ provided the resources. Both DZ and CT made equal contributions; DZ wrote the first draft and is listed first. All authors edited and commented on previous versions of the manuscript and approved the final version.

## Supplementary Material

Supplemental data

Supporting data values

## Version 1 12/06/2024

Electronic publication
