# Supplementary material for: Activation of connexin hemichannels enhances mechanosensitivity and anabolism in disused and aged bone
Source: JCI Insight. 2024 Dec 6;9(23):e177557. doi: 10.1172/jci.insight.177557 (PMC11623949; doi:10.1172/jci.insight.177557)
Supplement: Supplemental data [file jciinsight-9-177557-s128.pdf]

Supplemental Figure Legends

Figure S1. Cx43(M2) antibody enhances HC opening induced by mechanical loading in aged trabecular bone *in vivo*. Representative images of EB dye uptake in metaphyseal trabecular bone for both loaded and contralateral unloaded tibias of vehicle- and Cx43(M2)-treated mice. White arrowheads indicate EB-positive osteocytes. Scale bar, 20 μm.

Figure S2. Experimental setup for hindlimb suspension to mimic mechanical unloading. (**A**) Weekly monitoring of body weights in control and unloading mice of vehicle- and Cx43(M2)-treated mice. n=7-8 per group. (**B**) Schematic workflow of the experimental plan. (**C**) Copper wire used for HLS in a mouse tail. Data are expressed as mean ± SD. Statistical analysis was performed using two-way ANOVA with Tukey test among different groups (**C**).

Figure S3. Effect of enhanced activity of Cx43 HCs by Cx43(M2) on mechanical Mechanical properties during unloading and loading. (**A-D**) The three-point bending assay was performed for mid-diaphyseal femur bone of vehicle- and Cx43(M2)-treated mice: (**A**) stiffness, **(B)** elastic modulus, (**C**) ultimate force, and (**D**) ultimate stress. n=8 per group. (**E**) Mechanical loading did not change tibial stiffness in either vehicle- or Cx43(M2)-treated mice. n=6-7 per group. Data are expressed as mean ± SD. *, $P<0.05$; **, $P<0.01$; ***, $P<0.001$. Statistical analysis was performed using the paired t test for loaded and contralateral tibias (**E**), and two-way ANOVA with Tukey test for differences among groups (**A-E**).

Figure S4. Effect of Cx43M2 antibody on osteogenesis in control and unloading mice. (**A**) BFR/BS, (**B**) MAR, and (**C**) MS/BS were assessed along cortical surfaces of control and unloading

mice treated with either vehicle or Cx43(M2). (**D**) Representative images of double-calcein (green) labeling on tibias. n=3-6 per group. Scale bar: 50 μm. Data are expressed as mean ± SD. *, P<0.05. Statistical analysis was performed using two-way ANOVA with Tukey test among different genotypes (**A**-**C**).

Figure S5. Experimental setup for tibia axial compressive loading. (**A** and **B**) Schematic illustration of the experimental plan and tibia loading setup. (**C**) Diagram showing the left tibia positioned in the loading device and the direction of loading. (**D**) Weekly body weights of vehicle- and Cx43(M2)-treated groups. n=12 per group. Data are expressed as mean ± SD. Statistical analysis was performed using two-way ANOVA with Tukey test among different genotypes (**D**).

Figure S6. Cx43(M2) has no additional effect on cortical bone during mechanical stimulation in young adult mice. The cortical bone structure located 37% distal from the proximal end was analyzed in 16-week-old male mice loaded 5 days/week for 2 weeks. (**A**) Representative 3D models of the cortical bone in vehicle- and Cx43(M2)-treated mice. (**B**-**D**) μCT was used to assess structural parameters of cortical bone located 37% distal from the proximal end: (**B**) B.Ar, (**C**) B.Ar/T.Ar, and (**D**) Ct.Th. n=6-7 per group. Data are expressed as mean ± SD. *, P<0.05. Statistical analysis was performed using the paired t test for loaded and contralateral tibias (**B**-**D**), and two-way ANOVA with Tukey test for differences among groups (**B**-**D**).

Figure S7. Cx43(M2) antibody has no additional effect on load-induced endosteal osteogenesis in young adult mice. After 5 days/week loading for 2 weeks, bone histomorphometric analyses were performed on the tibias within cortical bone located 37% distal from the proximal end of 16-week-old vehicle- and Cx43(M2)-treated mice. (**A**) Representative

images of calcein (green) and alizarin (red) double labeling at the 37% diaphysis for all groups. Scale bar: 200 μm. (**B** and **E**) MAR, (**C** and **F**) MS/BS, and (**D** and **G**) BFR/BS were assessed along periosteal and endosteal surfaces of all tibias. n=3 per group. Scale bar: 200 μm. Data are expressed as mean ± SD. *, P<0.05; **, P<0.01; ***, P<0.001. Statistical analysis was performed using the paired t test for loaded and contralateral tibias (**B**-**G**), and two-way ANOVA with Tukey test for differences among groups (**B**-**G**).

Figure S8. **Cx43(M2) inhibits osteoclasts in tibial metaphyseal trabecular bone during mechanical loading.** (**A**) Representative images of TRAP-positive osteoclasts (red) in tibial metaphyseal trabecular bone. Scale bar, 80 μm. (**B**) Quantification of TRAP-positive osteoclast number per bone perimeter (N.Oc/BS) and (**C**) osteoclast surface per bone perimeter (Oc.S/BS) in tibial metaphyseal trabecular bone. n=5 per group. Data are expressed as mean ± SD. *, P<0.05. Statistical analysis was performed using the paired t test for loaded and contralateral tibias (**B** and **C**), and two-way ANOVA with Tukey test for differences among groups (**B** and **C**).

Fig. S1

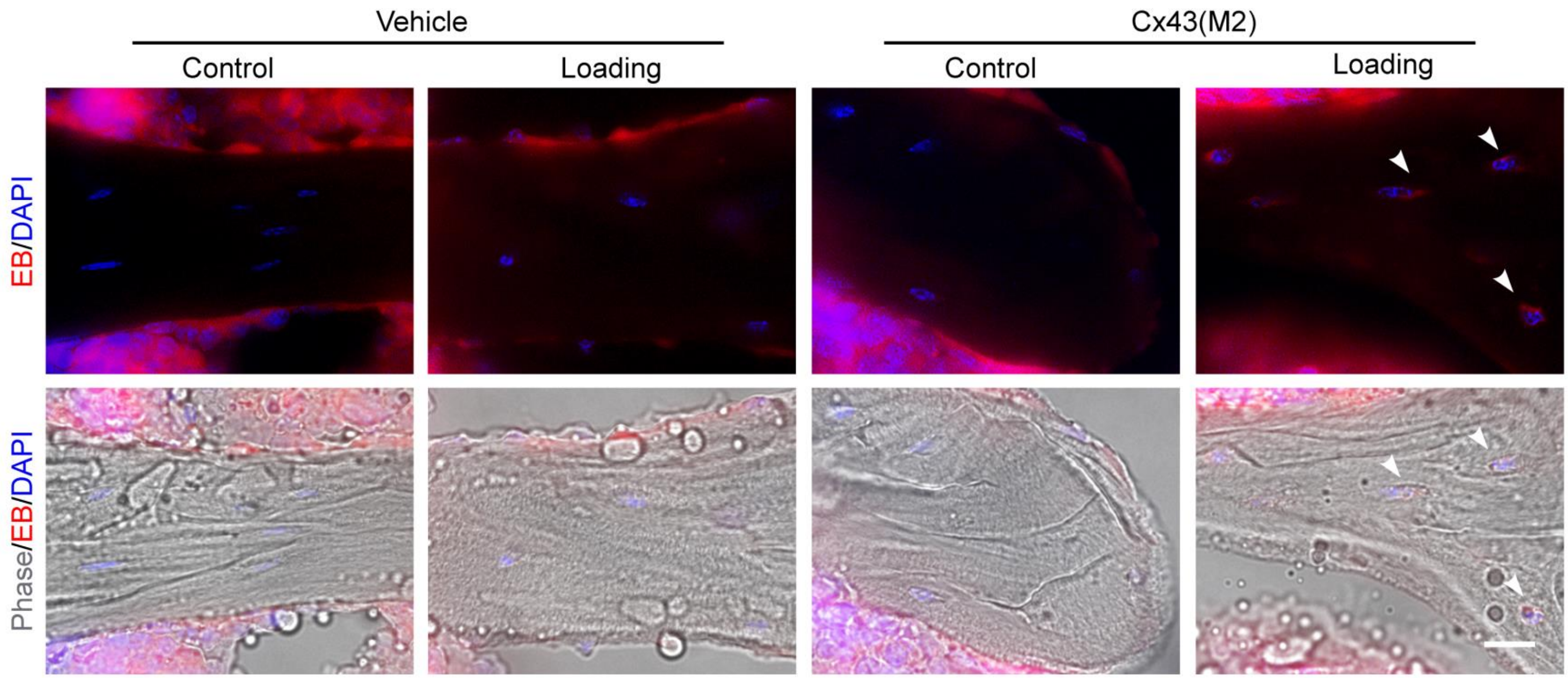

Vehicle
Cx43(M2)
Control
Loading
Control
Loading
EB/DAPI
Phase/EB/DAPI

Fig. S2

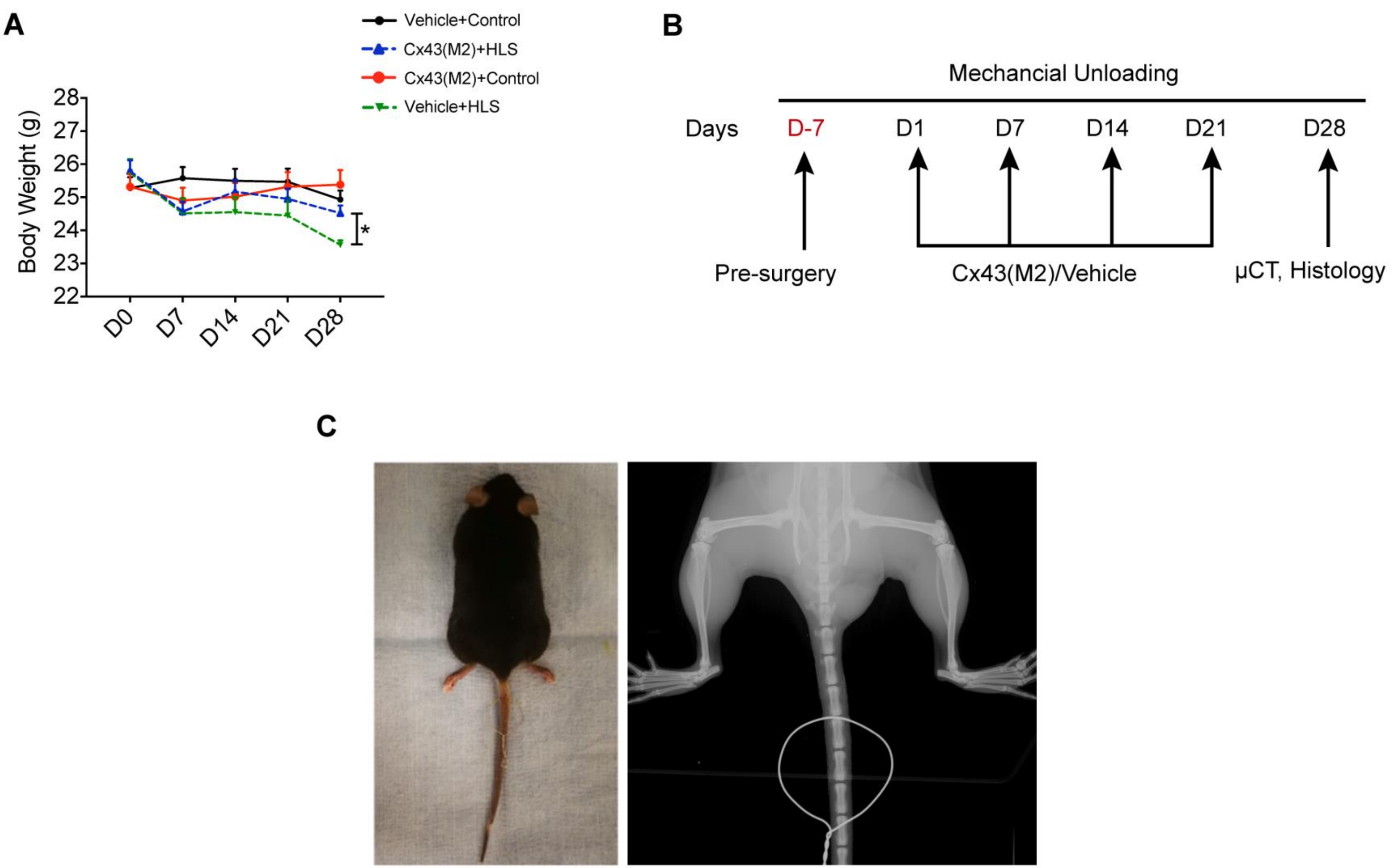
A
Vehicle+Control
Cx43(M2)+HLS
Cx43(M2)+Control
Vehicle+HLS
Body Weight (g)
28
27
26
25
24
23
22
*
D0
D7
D14
D21
D28
B
Mechancial Unloading
Days
D-7
D1
D7
D14
D21
D28
Pre-surgery
Cx43(M2)/Vehicle
μCT, Histology
C

Fig. S3

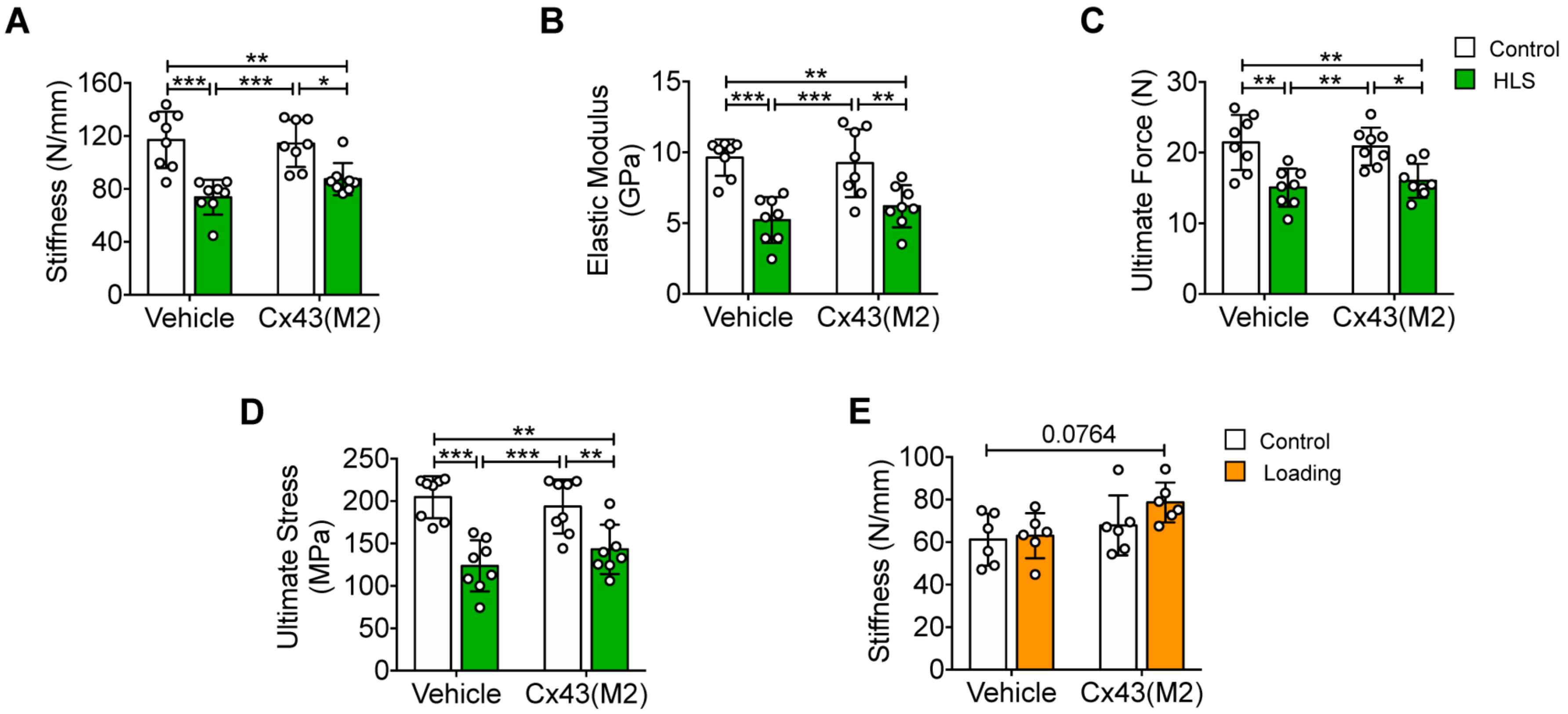
A
Stiffness (N/mm)
160
120
80
40
0
**
***
***
*
Vehicle
Cx43(M2)
B
Elastic Modulus (GPa)
15
10
5
0
**
***
***
**
Vehicle
Cx43(M2)
C
Ultimate Force (N)
30
20
10
0
**
**
**
*
Vehicle
Cx43(M2)
Control
HLS
D
Ultimate Stress (MPa)
250
200
150
100
50
0
**
***
***
**
Vehicle
Cx43(M2)
E
Stiffness (N/mm)
100
80
60
40
20
0
0.0764
Vehicle
Cx43(M2)
Control
Loading

Fig. S4

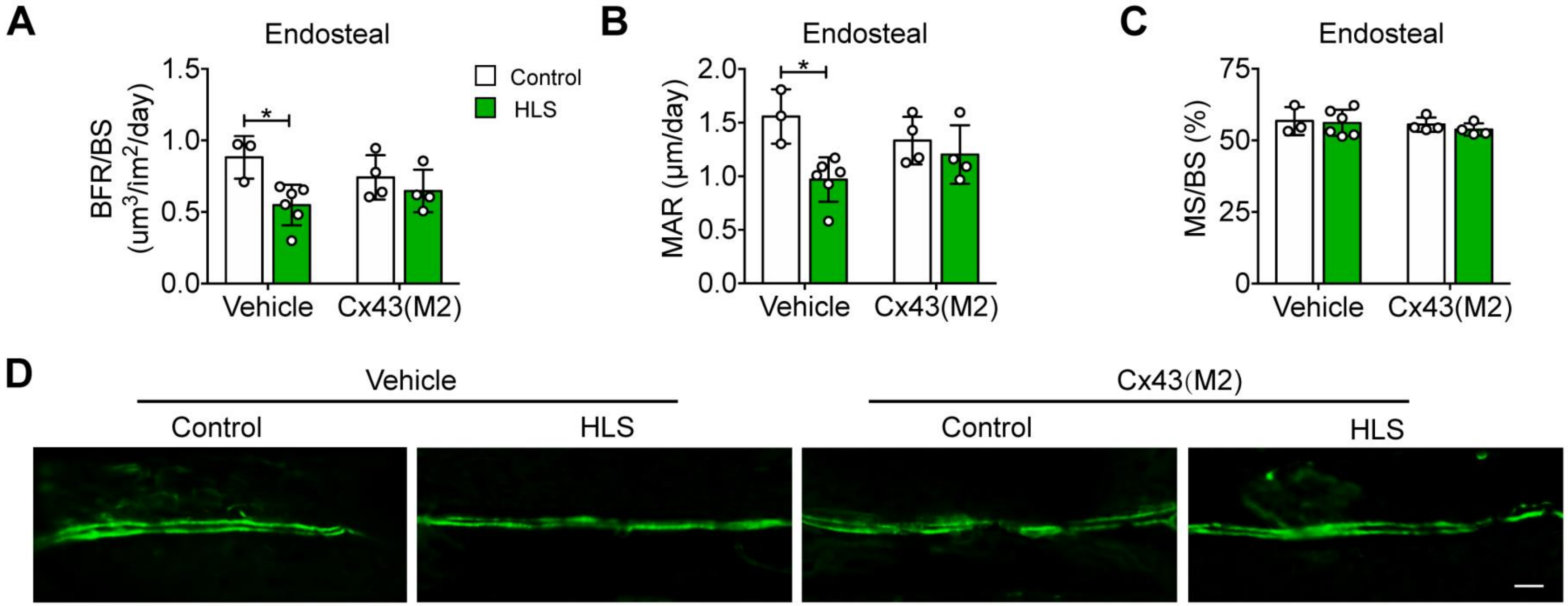
A
Endosteal
BFR/BS (um3/im2/day)
1.5
1.0
0.5
0.0
*
Control
HLS
Vehicle
Cx43(M2)
B
Endosteal
MAR (µm/day)
2.0
1.5
1.0
0.5
0.0
*
Vehicle
Cx43(M2)
C
Endosteal
MS/BS (%)
75
50
25
0
Vehicle
Cx43(M2)
D
Vehicle
Cx43(M2)
Control
HLS
Control
HLS

Fig. S5

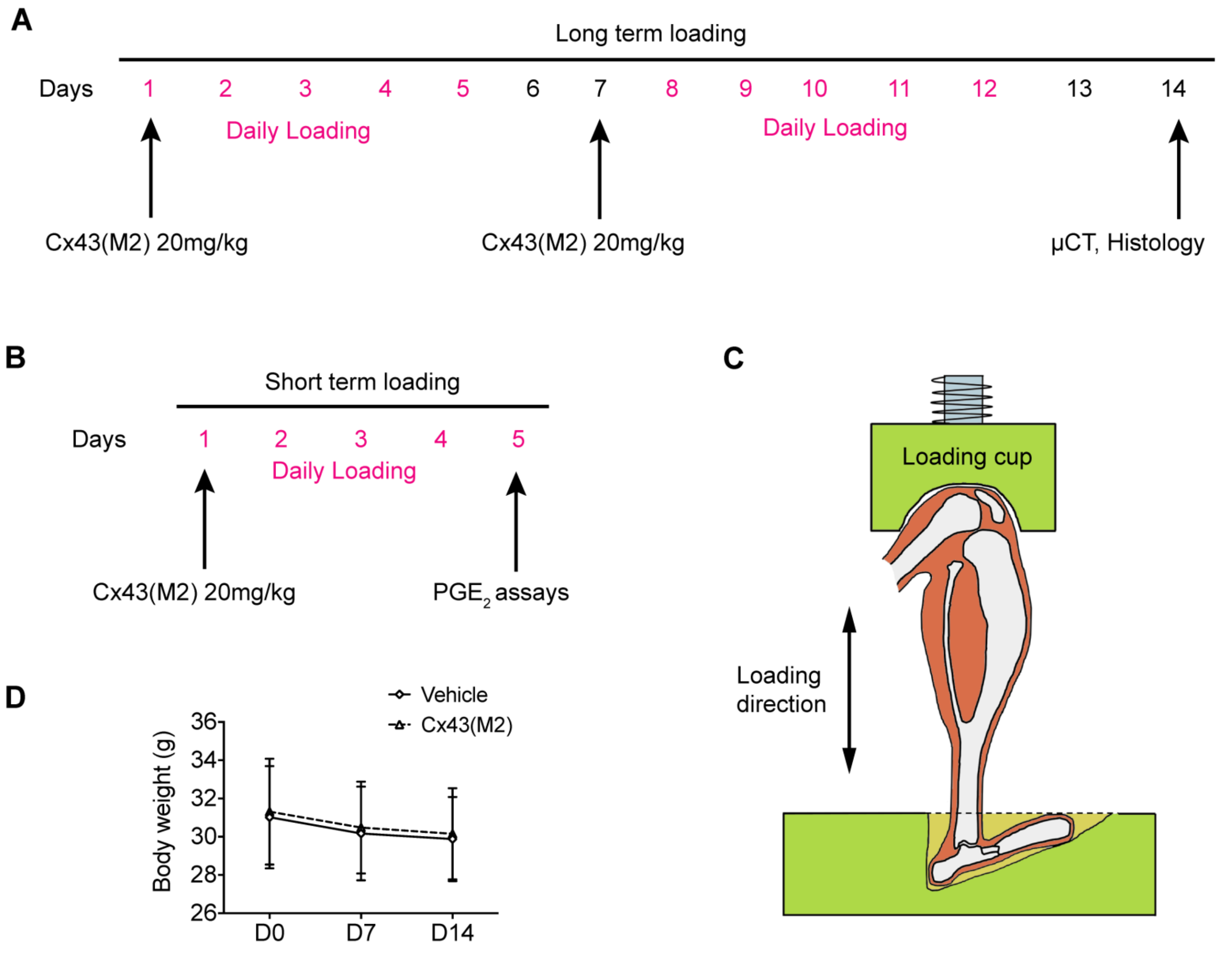
A
Long term loading
Days
1
2
3
4
5
6
7
8
9
10
11
12
13
14
Daily Loading
Daily Loading
Cx43(M2) 20mg/kg
Cx43(M2) 20mg/kg
μCT, Histology
B
Short term loading
Days
1
2
3
4
5
Daily Loading
Cx43(M2) 20mg/kg
PGE2 assays
C
Loading cup
Loading direction
D
Vehicle
Cx43(M2)
Body weight (g)
36
34
32
30
28
26
D0
D7
D14

Fig. S6

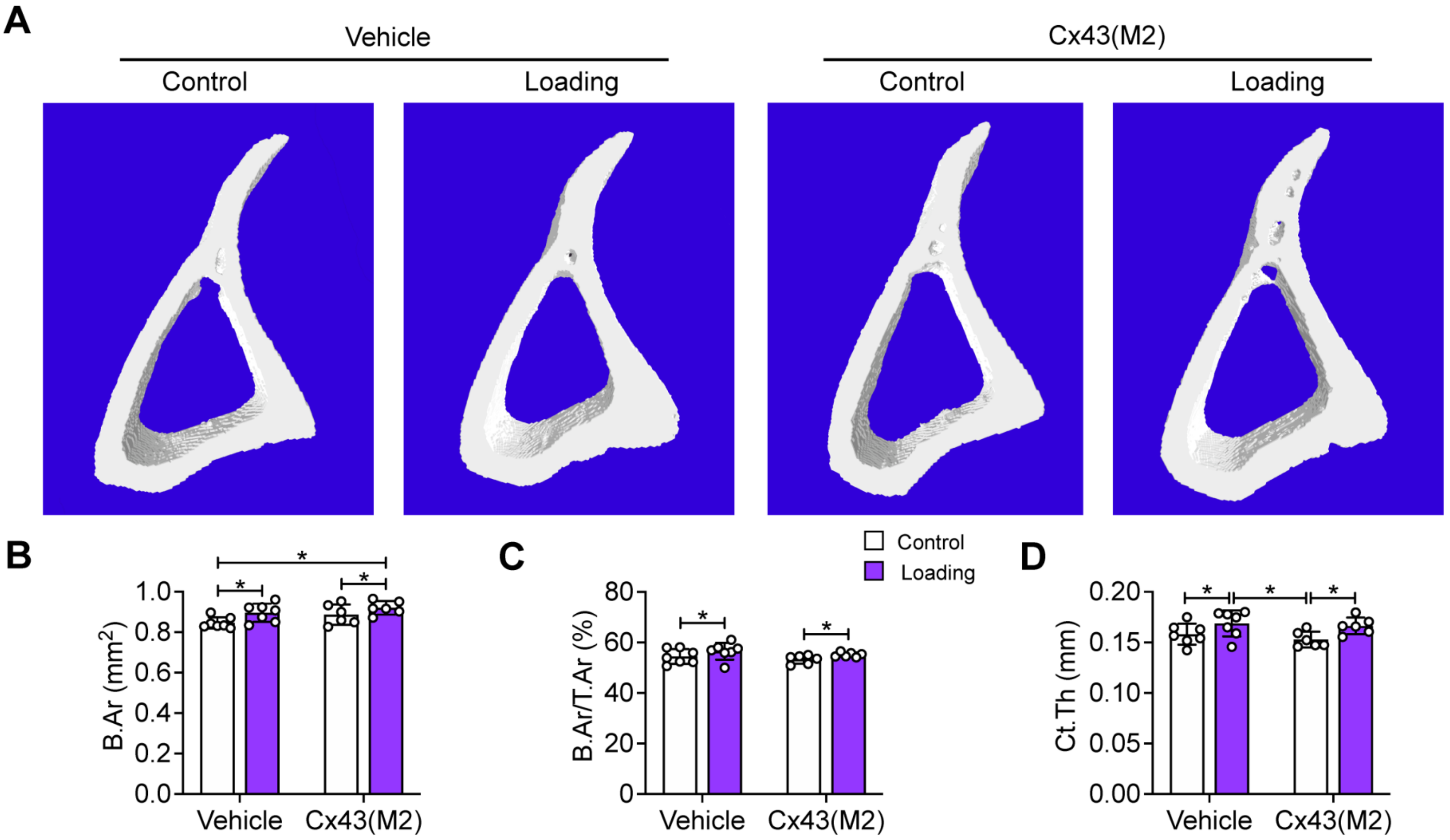
A
Vehicle
Cx43(M2)
Control
Loading
Control
Loading
B
C
D
Control
Loading
B.Ar (mm²)
1.0
0.8
0.6
0.4
0.2
0.0
*
*
*
Vehicle
Cx43(M2)
B.Ar/T.Ar (%)
80
60
40
20
0
*
*
Vehicle
Cx43(M2)
Ct.Th (mm)
0.20
0.15
0.10
0.05
0.00
*
*
*
Vehicle
Cx43(M2)

Fig. S7

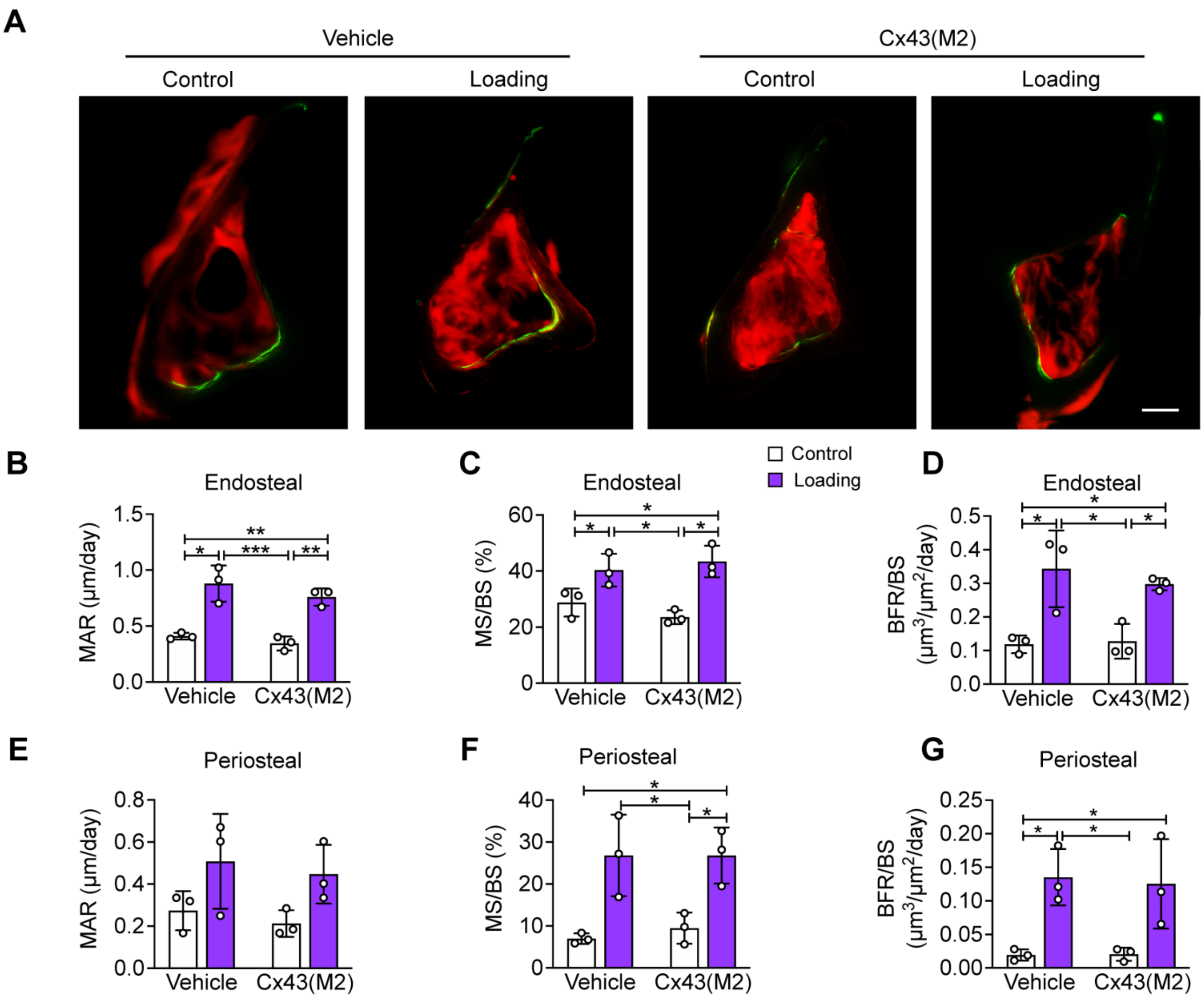
A
Vehicle
Cx43(M2)
Control
Loading
Control
Loading
B
Endosteal
MAR (µm/day)
1.5
1.0
0.5
0.0
Vehicle
Cx43(M2)
C
Endosteal
MS/BS (%)
60
40
20
0
Vehicle
Cx43(M2)
Control
Loading
D
Endosteal
BFR/BS (µm3/µm2/day)
0.5
0.4
0.3
0.2
0.1
0.0
Vehicle
Cx43(M2)
E
Periosteal
MAR (µm/day)
0.8
0.6
0.4
0.2
0.0
Vehicle
Cx43(M2)
F
Periosteal
MS/BS (%)
40
30
20
10
0
Vehicle
Cx43(M2)
G
Periosteal
BFR/BS (µm3/µm2/day)
0.25
0.20
0.15
0.10
0.05
0.00
Vehicle
Cx43(M2)

Fig. S8

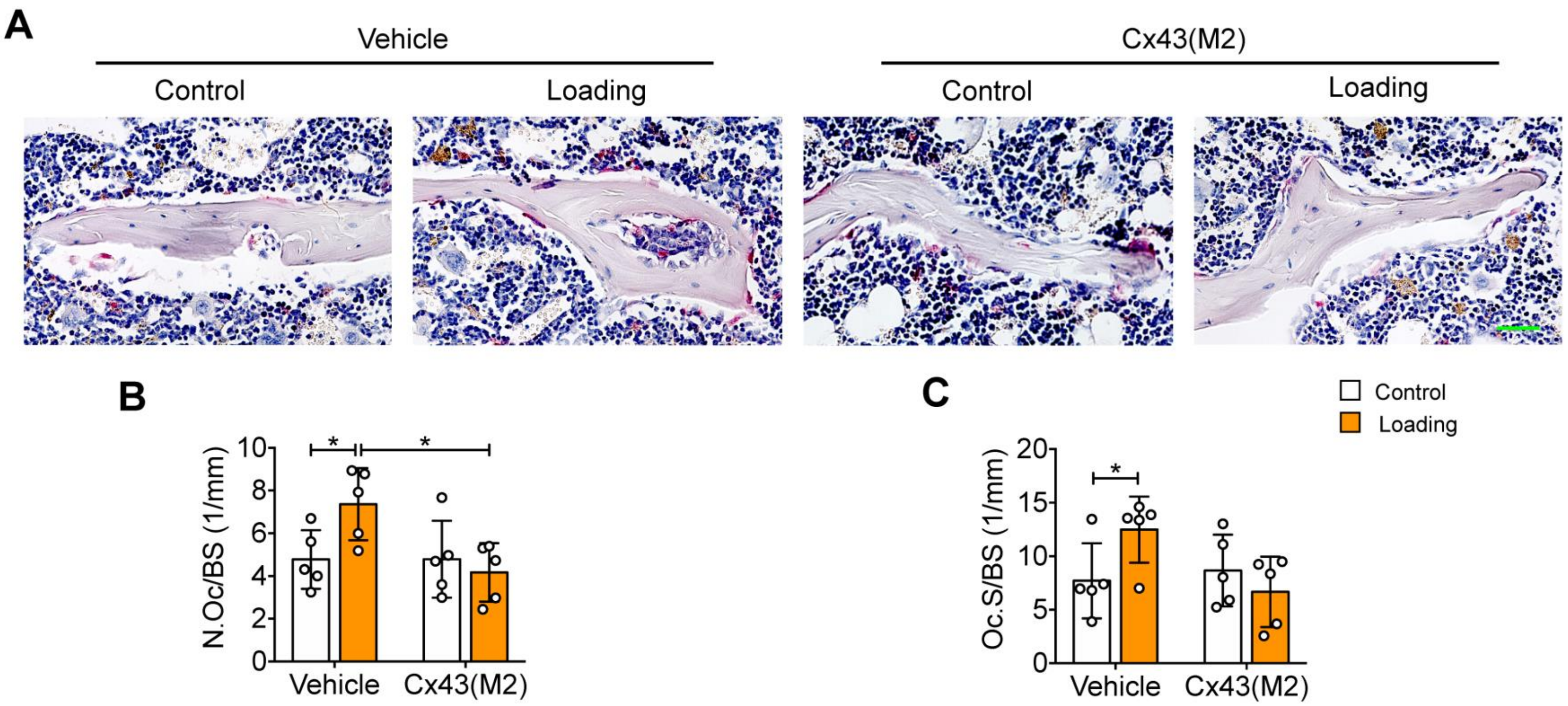
A
Vehicle
Cx43(M2)
Control
Loading
Control
Loading
B
N.Oc/BS (1/mm)
10
8
6
4
2
0
*
*
Vehicle
Cx43(M2)
C
Control
Loading
Oc.S/BS (1/mm)
20
15
10
5
0
*
Vehicle
Cx43(M2)
